# The Cat's Whiskers: Stable Isotopes Reveal Individual Specialisation of Adaptable Caracals (*Caracal caracal*) Foraging in an Urbanising Landscape

**DOI:** 10.1002/ece3.71154

**Published:** 2025-03-23

**Authors:** Gabriella R. M. Leighton, Anna R. Brooke, P. William Froneman, Laurel E. K. Serieys, Jacqueline M. Bishop

**Affiliations:** ^1^ Institute for Communities and Wildlife in Africa, Department of Biological Sciences University of Cape Town Cape Town South Africa; ^2^ Department of Biological Sciences University of Cape Town Cape Town South Africa; ^3^ Cape Leopard Trust Cape Town South Africa

**Keywords:** *Caracal caracal*, niche ecology, stable isotope analysis, trophic ecology, urban wildlife, whiskers

## Abstract

Urbanisation critically alters the availability of resources and the nature of risks for wildlife by fragmenting natural habitats and disrupting ecosystems. Despite these challenges, carnivores frequently persist in and around urban environments, where novel opportunities, such as anthropogenic food, may outweigh associated ecological risks. Here, we investigate the responses of an urban adapter to novel resources, using stable isotope analysis of vibrissae (whiskers) to understand the spatiotemporal foraging patterns of caracals ( *Caracal caracal* ) on the fringes of the city of Cape Town, South Africa. Caracals are medium‐sized felids and the largest remaining predators on the Cape Peninsula. Using isotopic niche metrics and home range estimates, we assess the effects of demographics, seasonality, and urbanisation on variation in individual foraging behaviour from GPS‐collar monitored caracals (*n* = 28) across an urban gradient. Despite a wide isotopic niche at the population level, we observed high levels of individual specialisation. Adult and male niches were wider, likely due to larger home ranges, which facilitate the exploitation of diverse prey across trophic levels. Patterns in *δ* ^13^C were seasonal, with increases during the warmer, drier summer months across the Peninsula irrespective of habitat use. Taken together with niche contraction for caracals in urban areas, our findings suggest higher reliance on human‐subsidised resources in summer. Caracals using areas dominated by wildland cover had higher *δ* ^15^N values and larger niches than those using urban‐dominated areas. Across the study area, *δ* ^15^N values varied spatially, with increased enrichment in caracals using more coastal and wetland areas and prey, particularly in winter. Individual foraging flexibility in caracal is clearly a key strategy for their success in this rapidly transforming landscape. Understanding spatiotemporal shifts in dietary niche and trophic ecology in adaptable urban carnivores, like the Cape Peninsula caracals, is fundamental for understanding the ecological needs of wildlife in and around rapidly growing cities.

## Graphical abstract

We use stable isotope analysis of sequential whisker segments to investigate the responses of an urban adaptor to novel resources in and around a major city in the Global South. The caracal, an adaptable mid‐sized wild cat species, alters trophic foraging ecology depending on demographics, season and level of exposure to urbanisation in Cape Town, South Africa. Caracals have wide isotopic niches at a population level, but intraindividual variation reveals individual specialisation, revealing that spatiotemporal shifts and individual foraging flexibility are key to their persistence in rapidly transforming landscapes.

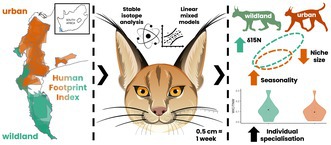

## 1 Introduction

Resources and risks around cities are substantially altered in space and time (McKinney 2002; Sih et al. 2011). The resilience of wildlife in urban landscapes is mediated by their ability to respond to these changes in resources while minimising exposure to associated risks (Lowry et al. 2013; Newsome et al. 2015b; Mazza et al. 2020; Suraci et al. 2021). Although selective urban pressures can have contrasting effects among mammalian species, behavioural flexibility in mammals allows them to better adapt to urban environments (Santini et al. 2019; Ritzel and Gallo 2020). Generally, mammals are easily disturbed by human activities, which drive changes in their behaviour that can impact diet, reproduction, stress, activity budgets and disease prevalence (Ditchkoff et al. 2006; Birnie‐Gauvin et al. 2016; Ritzel and Gallo 2020). These adaptive responses may scale up to impacts on ecosystem function (Alberti 2015). Apart from differences between species, individual variation in behavioural flexibility may arise from developmental constraints, sex, age or personality (Bókony et al. 2012; Holekamp et al. 2013; Vardi and Berger‐Tal 2022). For example, within a species, female and male individuals may adjust their home ranges and resource use patterns differently when adapting to urban environments (Atiqah et al. 2015; Ritzel and Gallo 2020). Variation within ‘urban adapters’ (*sensu* McKinney 2002) can result in specialisation at an individual level, even within populations of ecologically generalist species (Bolnick et al. 2002, 2003, 2011). In this way, generalists may reduce intraspecific competition in urbanising areas through temporal and spatial resource partitioning. For example, populations of generalist red foxes ( *Vulpes vulpes* ; Scholz et al. 2020) and coyotes ( *Canis latrans* ; Newsome et al. 2015a) demonstrate individual diet specialisation in urban areas, which likely facilitate territory sharing in space‐limited human‐transformed environments.

Behavioural flexibility in urban systems is largely influenced by changes in environmental stability and predictability, where stable habitats may favour less flexible species or individuals (Vardi and Berger‐Tal 2022). Cities are constantly changing habitats for wildlife but often provide increased resources for species that take advantage of the availability of anthropogenic food sources, shelter and novel prey species (Bateman and Fleming 2012; Fleming and Bateman 2018). Mammalian carnivores, for example, may adjust their behaviour to avoid human activity and thereby capitalise on urban resources; as a result, their diets frequently track resources within the urban landscape (Bateman and Fleming 2012; Lowry et al. 2013; Riley et al. 2021). In doing so, the year‐round availability of both natural and anthropogenic food resources within urban spaces may buffer the fluctuations and ecological effects that characterise ‘natural’ resource availability alone (e.g., seasonal fluctuations; Shochat 2004; Vardi and Berger‐Tal 2022), thereby altering the ecological interactions between urban‐adapted predator and prey populations (Newsome et al. 2015b). Depending on the season and resource availability within an urban landscape, regular prey‐switching can occur between natural and human‐associated prey, resulting in a high degree of inter‐individual variation in diet (Newsome et al. 2015b). Ultimately, the benefits of resource‐rich human‐impacted environments can outweigh the associated costs for many species and significantly alter the trophic interactions of carnivores in urban areas.

Studying shifts in the seasonality of diet and individual dietary specialisation in urban areas can improve our understanding of changing predator–prey relationships over time in rapidly developing landscapes (Bolnick et al. 2011; Moss et al. 2016; Scholz et al. 2020). To do this, stable isotope analysis (SIA) of inert tissue, such as fur and vibrissae (whiskers), provides insight into integrated diet, movement and trophic interactions over longer time periods (Kernaléguen et al. 2012; McHuron et al. 2016; Rogers et al. 2020). Higher *δ* ^13^C isotope ratios usually indicate that the prey species consumed by an individual have higher proportions of plants with C_4_ photosynthetic pathways, such as corn ( *Zea mays* ) and sugarcane (*Saccharum spp*.), which are common in anthropogenic food sources (Jahren et al. 2008), while lower values are predominantly associated with C_3_ plants found in natural habitats (Gámez et al. 2022). Elevated *δ* ^15^N isotope ratios indicate the consumption of protein‐enriched prey species at higher trophic levels, particularly marine species which tend to be part of longer food chains (West et al. 2006), providing information regarding the trophic status of the consumer (Ben‐David and Flaherty 2012; Pollard and Puckett 2021). When investigating mammalian diet, whiskers are particularly useful, providing a valuable chronological record of the foraging ecology and habitat use of individuals, and tracking monthly and thus seasonal, isotopic changes of an individual animal (e.g., Kernaléguen et al. 2012; Voigt et al. 2018; McHuron et al. 2019; Scholz et al. 2020; Attard et al. 2021).

**FIGURE 1:**
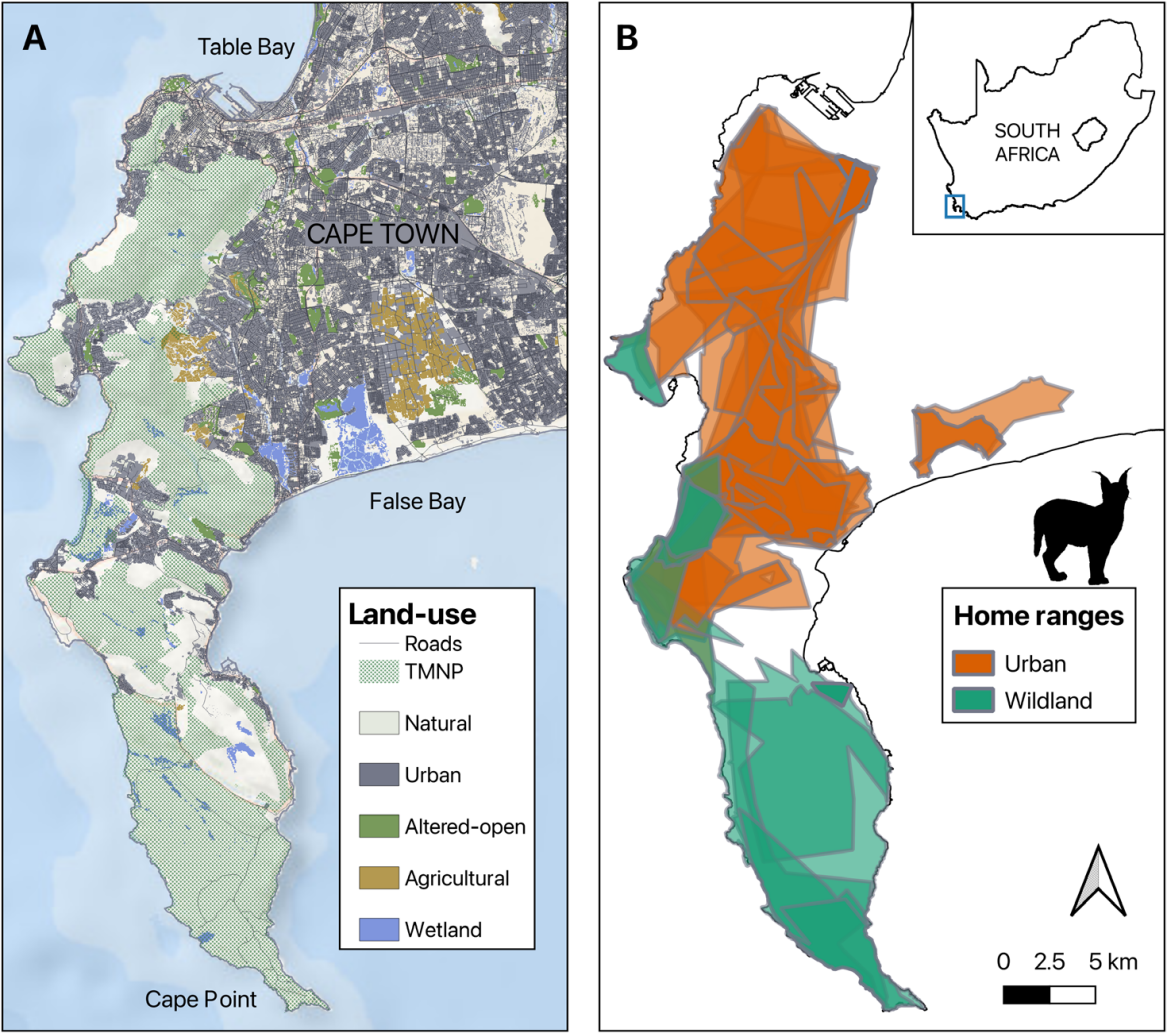
Study area map coloured by land‐use with roads in grey (**A**) and 95% t‐LoCoH home ranges of monitored caracals on the Cape Peninsula (**B**). Urban‐dominated home ranges are in orange (*n* = 18) and wildland‐dominated home ranges (*n* = 10) are in green as classified by the Human Footprint Index.

Caracals ( *Caracal caracal* ) are elusive, medium‐sized (6–20 kg) predators that are highly generalist, obligate carnivores that exploit a wide variety of small and mid‐sized mammals, rodents, birds, reptiles, and insects across their range (Avenant and Nel 2002; Leighton et al. 2020; Parchizadeh et al. 2023). Following the local extinction of Cape leopards ( *Panthera pardus pardus* ) by the 1930s and, before that, Cape lions ( *P. leo melanochaita* ) by the mid‐19th century, caracals are the largest remaining predators of the Cape Peninsula (Figure 1A), which is spatially isolated by the city of Cape Town, South Africa (Anderson and O'Farrell 2012). Here, caracals have successfully adapted to exploit the coastal and urban edges and remaining fragments of wildland areas (Leighton et al. 2022b; Serieys et al. 2023). In this study, we explore the dietary flexibility in Cape Town's caracal to better understand how these mammalian carnivores can succeed and potentially persist in rapidly urbanising landscapes, building on work from a long‐term research effort. Previous work on caracal foraging ecology using SIA of fur from monitored and unknown individuals reveals important effects of human and marine subsidies on trophic and niche dynamics at a larger scale (e.g., Leighton et al. 2023, 2024). Here, we focus on the comparative effects of demographics, seasonality, and urbanisation on variation in the foraging ecology of a smaller subset of monitored caracals only on the Cape Peninsula, using SIA of whiskers as chronological records of their diet. Using detailed movement data from GPS collars and the analysis of *δ* ^13^C and *δ* ^15^N isotope ratios from whisker segments, we (i) assess the relative influence of caracal demographics (age and sex group) and seasonality on changes in foraging patterns based on *δ* ^13^C and *δ* ^15^N values, (ii) test for evidence of individual niche specialisation and whether the level of this specialisation changes with exposure to urbanisation, and (iii) evaluate the effects of spatial habitat use and diet on whisker *δ* ^13^C and *δ* ^15^N values.

## 2 Methods

### 2.1 Ethics Statement

All animal capture, handling and sample collection followed ethical guidelines approved by the American Society of Mammologists, the University of Cape Town Animal Ethics Committee (2014/V20/LS), Cape Nature (AAA007‐0147‐0056) and South African National Parks (SERL/AGR/017–2014/V1).

### 2.2 Study Area

Samples were collected from caracals using the Cape Town area in the Western Cape province of South Africa (Figure 1A). The Cape Peninsula is spatially isolated from the rest of the country by the urban sprawl of Cape Town (Figure 1A). It is a fragmented mosaic of mostly wildland habitat that makes up the Table Mountain National Park (TMNP), with approximately 320km^2^ of available habitat. Here we define ‘wildland’ as natural areas and also including areas that are not formally protected within the TMNP, such as altered‐open areas, and open agricultural areas (e.g., vineyards and pine plantations that are accessible to wildlife; see Figure 1A). In the North, TMNP is predominantly bordered by urban development; however, in the south the park is surrounded by ocean and less urban development (Figure 1A). The climate is Mediterranean with a period of summer drought and mean annual rainfall of 600–800 mm/y mainly falling in the cold, wet winter months (Cowling et al. 1996). The past three decades have seen a significant increase in Cape Town's population (~46% increase between 1996 and 2011; Allsopp et al. 2014), the degree of urbanisation, and subsequent habitat fragmentation.

### 2.3 Sample Collection

We used standard cage‐trapping techniques to capture caracals between 2014 and 2016 as part of a long‐term study by the Urban Caracal Project (detailed capture and collaring method described in Serieys et al. 2023 and on Serieys and Bishop 2024). Briefly, individuals were fitted with GPS collars (Followit Tellus, Lindesberg, Sweden) with a release mechanism and rot‐off cotton spacer to ensure eventual drop‐off. Collars recorded at two fix intervals: every 3 h, and on every 9–10th day at 20‐min intervals for 24–36 h, for a maximum of 6 months. For each individual we recorded age class, sex, weight, and morphological measurements and took fur and whisker samples. Individuals were classified as subadults (< 2 years) or adults (≥ 2 years) based on body size, weight, tooth wear and eruption, and reproductive status (Schroeder et al. 2005). The new whisker dataset analysed here represents 28 unique individual caracals, some of which (*n* = 11) were sampled more than once due to recaptures or subsequent mortality sampling. One male was collared twice, once as a subadult (TMC16a) and again 10 months later as an adult (TMC16b; Figure S1) and is therefore considered two separate ‘individuals’. The whiskers prioritised for analysis were those for which we had GPS data of the individual caracal, where the whisker represented growth during the collaring periods, allowing the isotopic data to be linked with location data by date. Whiskers were collected from live individuals under sedation during capture for GPS‐collaring (*n* = 25 caracals), and from post‐mortem individuals (*n* = 10 caracals; e.g., roadkill mortalities). Following Mutirwara et al. (2018) the longest whisker sampled for each animal was selected to standardise the whisker position and to allow the longest period of diet assimilation for comparative analysis.

### 2.4 Growth Rate Calculations

No whisker growth rate data is currently available for caracals; therefore, growth rates measured for captive large felid whiskers (i.e., lions and leopard; Mutirwara et al. 2018) were used to estimate the growth rate. The study provides the only known data on growth rates for wild felids in South Africa and is therefore more appropriate than domestic cat estimates in the Global North (e.g., Cecchetti et al. 2021). Felid whisker growth rate is non‐linear (Mutirwara et al. 2018); however, the small sample size in this study meant there was not sufficient statistical power to estimate parameters for a non‐linear model; thus, linear growth rates were assumed. Starting from the root, each whisker was cut into 5 mm sections, which was equivalent to approximately 1 week of growth based on an average growth rate of 0.65 ± 0.01 mm.day^−1^ (Mutirwara et al. 2018). To estimate the dates represented by each whisker section, the mean, maximum (0.84 mm.day^−1^) and minimum (0.50 mm.day^−1^) growth rate estimates were used, where the growth time for each section (to the nearest day) was subtracted from the whisker collection date (see Figure S1). Based on these dates, the season (winter vs. summer) was calculated by assigning dates in the summer and spring months (September—February) to summer and dates in the winter and autumn months (March—August) to winter (Figure S1).

### 2.5 Sample Preparation and Stable Isotope Analysis

Sectioned whiskers were degreased for 24 h using a cleaning solution of chloroform, methanol, and distilled water in a ratio of 2:1:0.8, as described in Bligh and Dyer (1959) and following Lee‐Thorp et al. (1989). The whisker segment was then rinsed with 1 mL distilled water three times and dried at 40°C for at least 24 h in a drying oven (Lasec Series 2000). Cleaned samples were processed in the Archaeology Department's Isotope Laboratory at the University of Cape Town. The whisker samples were weighed (0.3–0.5 mg) using a microbalance (Sartorius, model M2P micro balance), where more distal sections were combined to make up the weight if required, before being folded into tin cups to 1 mcg accuracy. These were then combusted in a Delta V Plus organic elemental analyser/isotope ratio mass spectrometer (IRMS) via a Conflo IV gas control unit (Thermo Scientific, Germany). The calibrated standards used by both laboratories were DL Valine, Merck Gel, sucrose and Choc, which are calibrated against International Atomic Energy Agency (IAEA) standards. Nitrogen is expressed relative to atmospheric nitrogen; carbon is expressed relative to Pee‐Dee belemnite (Ben‐David and Flaherty 2012).

### 2.6 Spatial and Dietary Variables

The *tlocoh* R package was used to calculate 95% local convex hull (LoCoH) home range estimates for all monitored individuals (see Leighton et al. 2022b for details). The T‐LoCoH *a* method accounts for areas with different intensities of use and is appropriate for movement data that is influenced by irregular hard boundaries (Getz et al. 2007), such as urban and coastal edges. The multistep approach outlined by Lyons et al. (2013) was followed to optimise parameters (i.e., number of nearest neighbours and hull‐sets to choose the best *a*‐value) per individual. One adult male (TMC33) was ear‐tagged so he could be identified, but he was not collared. A T‐LoCoH home range was therefore estimated using sightings data received from the public over 5 years, likely providing representative data to estimate a reasonable home range (*n* = 148, August 2018—May 2023).

Previous work on the population shows that caracals on the Peninsula adjust their foraging behaviour based on their level of exposure to urbanisation (Leighton et al. 2022b). Therefore, individuals were classified based on their mean Human Footprint Index (HFI) values within their home ranges as using either urban‐dominated or wildland‐dominated areas (Figure 1B). We calculated the mean HFI 2018 release (a score from 0 to 50; Venter et al. 2016, 2018) within each home range. The Human Footprint Index is an effective proxy for human development and disturbance as it incorporates built environments, human population density, electric infrastructure, crop lands, pasture, roads, railways, and navigable waterways into a single metric at a global scale (Venter et al. 2016, 2018). Home ranges with < 25 mean HFI were classified as ‘wildland‐dominated’ while other home ranges (≥ 25; i.e., half the HFI score) were classified as ‘urban‐dominated’ following Leighton et al. (2023). According to this HFI classification, wildland areas are those outside of the city, with low population density and small settlements with or without agricultural activity. This resulted in 18 individuals with urban‐dominated home ranges and 10 individuals with wildland‐dominated home ranges (range 11.96–38.33 HFI; see Figure 1B). Additional variables considered were proportion urban and wetland area, using the 2018 Department of Environmental Affairs (DEA) national land‐cover raster following Leighton et al. (2022a). Binary rasters were created for each land‐use type, and the proportion of each land‐use variable within each estimated home range was calculated. Additionally, we calculated the mean distance from the coast within each estimated home range (range: 0.39–5.39 km).

To investigate how seasonality in diet potentially influences isotope signatures in caracal, we used a previously analysed, detailed dietary dataset (Leighton et al. 2020), which integrates both scat and caracal feeding sites located at GPS clusters of collared individuals on the Cape Peninsula. Diet proportions were based on the biomass of prey groups per caracal individual. Details of biomass calculations are in Leighton et al. 2020. Briefly, we calculated proportion edible mass using proportions scaled to equivalent prey mass categories (adapted for caracals from Funston et al. 1998, Appendix S1) for found prey remains, and the generalised felid biomass model developed by Chakrabarti et al. (2016) for prey items identified in scat. Dietary patterns were assessed by prey foraging habitat type (i.e., predominantly in terrestrial, wetland, arboreal and marine environments).

### 2.7 Statistical Methods

To assess whether the categorical variables of caracal sex, age, exposure to urbanisation, or seasonality affect isotopic values, linear mixed models (LMMs) were used to test for differences in the *δ* ^13^C and *δ* ^15^N values between various caracal groupings. The full models included sex (male vs. female), age (adult vs. subadult), home range type (wildland‐ vs. urban‐dominated), and binary season (winter vs. summer; see Table 1). A random effect term of the caracal individual accounted for pseudo‐replication, as there were multiple whisker segments per individual. For this first set of LMMs with categorical variables, we examined the full models (see Table 1).

**TABLE 1:** Full model structure for linear mixed models (LMMs) of *δ* ^13^C and *δ* ^15^N values investigating the potential influence of season and *Caracal* groups (age, sex and urban‐ vs. wildland‐dominated home ranges), the effect of seasonal impacts on spatial variables, and the effect of seasonal impacts on dietary variables (*n* = 6 models in total).

Variable	*δ* ^13^C/*δ* ^15^N season + caracal groups (*n* = 244)	*δ* ^13^C/*δ* ^15^N season + spatial (*n* = 244)	*δ* ^13^C/*δ* ^15^N season + dietary (*n* = 202)
Age (adult vs. subadult)	•		
Sex (male vs. female)	•		
Home range type (urban‐ vs. wildland‐dominated)	•		
Season (summer vs. winter)	•		
Demographic (adult male, adult female, subadult male, subadult female)		•	•
Season × proportion wetland area		•	
Season × proportion urban area		•	
Season × coastal distance		•	
Season × terrestrial biomass			•
Season × arboreal biomass			•
Season × marine biomass			•
Season × wetland biomass			•
Random effect: caracal ID	•	•	•
Random effect: home range type		•	•

Isotopic niche metrics were calculated and plotted based on Bayesian ellipses of *δ* ^13^C and *δ* ^15^N recorded in each individual whisker segment using the *SIBER* package in R. These multivariate ellipse‐based metrics allowed comparison of the isotopic niche width and overlap of demographic groups and seasons. Standard 95% ellipse areas (SEA) were estimated to assess the isotopic niche widths following correction for small sample sizes (SEAc). We compared differences in the total area (TA), mean SEAc, and overlap of demographic groups (males and females, and adults and subadults), level of exposure to urbanisation (urban‐ vs. wildland‐dominated) and season (summer vs. winter).

Next, using the unique temporal information from the whisker segments, we investigated the effect of several spatial and dietary variables on caracal isotope signatures and how this may be mediated by season using another two sets of LMMs (see Table 1). We were interested in exploring both spatial and dietary variables; thus, to avoid collinearity and overfitting, we ran separate models (Hawkins 2004; Tredennick et al. 2021). First, for the spatial models, we investigate the importance of urbanisation in buffering caracal diet from natural seasonal changes. LMMs assessed how the predictor variables of season (winter vs. summer), proportion of urban area in home range, proportion of wetland in home range, and mean distance to coast within home range influenced both *δ* ^13^C and *δ* ^15^N signatures (response variables) in whiskers. The full models included a categorical variable for demographic (adult male, adult female, subadult male and subadult female), interaction terms between season and proportion of urban area; season and proportion of wetland area; and season and coastal distance for *δ* ^13^C and *δ* ^15^N (Table 1), to investigate if potential seasonal changes in diet are moderated by caracal spatial habitat use. For the spatial LMMs, home range type (urban‐ vs. wildland‐dominated) was included as a random effect, as there are known differences in foraging between these habitats (Leighton et al. 2022b) and we wanted to investigate overall patterns at a Peninsula level. Then, for the diet LMMs, we investigate the influence of dietary and temporal variables on isotope patterns for those individuals for which we had dietary information. The full models for dietary LMMs included demographic and interactions between season and the proportion of terrestrial, arboreal, marine, and wetland‐adapted prey biomass in caracal diet (Table 1). A random effect term of caracal individual was included in all LMMs. For these spatial and dietary LMMs, we selected the top models using AICc (Burnham and Anderson 2002) by fitting models with all covariate permutations using the *MuMin* package (Bartoń 2020). Collinearity between all variables was assessed with variance inflation factors (VIF < 3).

To test if the level of individual specialization changes based on exposure to urbanization and use of prey subsidized by human resources, we calculated the within individual variation/total niche width (WIC/TNW) ratio following Scholz et al. (2020) and as suggested by Bolnick et al. (2002). Following this ratio, a foraging specialist can be defined as an individual whose dietary niche width is narrower than the total niche width of the population (TNW; Bolnick et al. 2002; Scholz et al. 2020). The TNW comprises within individual (WIC) variation in resource use, as well as between individual (BIC) variation. The TNW value is represented by the total ellipse area (TA) of the trophic niches represented by caracals with urban‐ and wildland‐dominated home ranges, and the WIC is represented by the TA of each individual. A TNW/WIC ratio value approaching 1 suggests that individuals are generalists and use most of the available niche of the population, while smaller values indicate individual dietary specialization (Scholz et al. 2020). All statistical analyses were conducted using R v. 4.4.1 (2024‐06‐14) (R Core Team and Team 2024). Plots were created in R using *SIBER* and *ggplot2*, and the map was created using QGIS v. 3.34.9 (QGIS Development Team 2024).

## 3 Results

**FIGURE 2:**
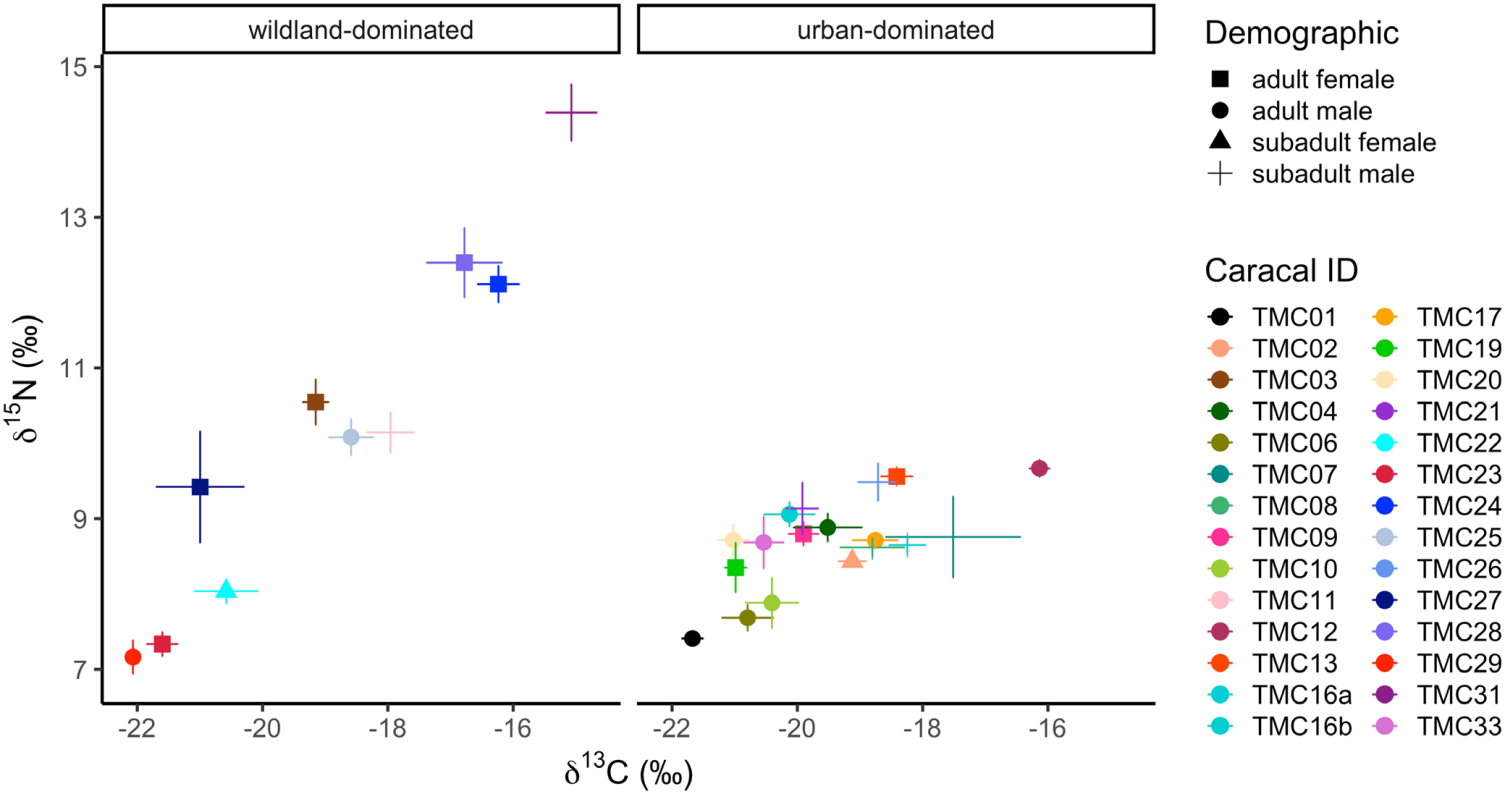
Mean ± SE of *δ* ^15^N and *δ* ^13^C isotope values in whiskers sampled from caracals with wildland‐ and urban‐dominated home ranges on the Cape Peninsula, South Africa. Data points are colored by caracal individual and shaped by demographic group.

The caracal whiskers (*n* = 41) from 28 unique individuals were on average 67.81 ± 15.93 mm long. Based on felid growth rates from the literature (i.e., Mutirwara et al. 2018), whiskers represented on average 104.32 days (3.43 months). However, because whiskers thin towards the distal end, on average, each whisker produced only 6.11 ± 1.62 usable segments (i.e., segments with enough material to weigh into the capsule for SIA). Therefore, caracal whiskers represented only 47 (34–59) days (1.11–1.94 months; see Figure S1). Caracal whiskers sampled across the study area showed a wide range of both *δ* ^15^N (mean = 9.28‰ ± 1.89‰, range = 5.54‰ to 16‰) and *δ* ^13^C (mean = −19.29‰ ± 1.81‰, range = −22.62‰ to −13.37‰) values (Figure 2; Table 2). The whisker samples capture caracal flexibility, showing individual variation in isotope values but within the range of fur samples analysed previously (Leighton et al. 2023; see Figure S2 for comparison of fur and whisker values). There was decreased variance in both *δ* ^13^C and *δ* ^15^N values of caracals with urban‐dominated compared to wildland‐dominated home ranges (Figure 2; Table 2). Caracal home ranges differ significantly depending on demographics, where male home ranges are larger than females (sex: *F*_1_ = 11.40, *p* < 0.01; Table S1), and adult home ranges are larger than subadults (age: *F*_1_ = 5.69, *p* < 0.05; Table S1).

**TABLE 2:** Mean ± SD, variance, and range of raw *δ* ^15^N and *δ* ^13^C isotope values of whisker segments from caracals on the Cape Peninsula, South Africa.

Group	*n*_caracals_	*n*_whisker segments_	Mean *δ* ^15^N	Variance *δ* ^15^N	Range *δ* ^15^N	Mean *δ* ^13^C	Variance *δ* ^13^C	Range *δ* ^13^C
Wildland	10	94	10.41 ± 2.44	5.97	6.12–16.00	−18.59 ± 2.58	6.64	−22.63 – −13.37
Urban	18	150	8.58 ± 0.88	0.77	5.54–10.98	−19.68 ± 1.69	2.85	−22.58 – −15.01
Female	10	83	9.63 ± 1.77	3.12	6.95–14.03	−19.11 ± 1.91	3.67	−22.43 – −14.21
Male	18	161	9.11 ± 1.93	3.72	5.54–16.00	−19.34 ± 2.25	5.04	−22.63 – −13.37
Adult	19	176	9.00 ± 1.63	2.66	5.54–14.03	−19.70 ± 2.04	4.15	−22.63 – −14.21
Subadult	9	68	10.01 ± 2.29	5.22	7.51–16.00	−18.12 ± 1.97	3.88	−21.36 – −13.37

### 3.1 Caracal Group and Seasonality Effects on Isotopic Signature

**FIGURE 3:**
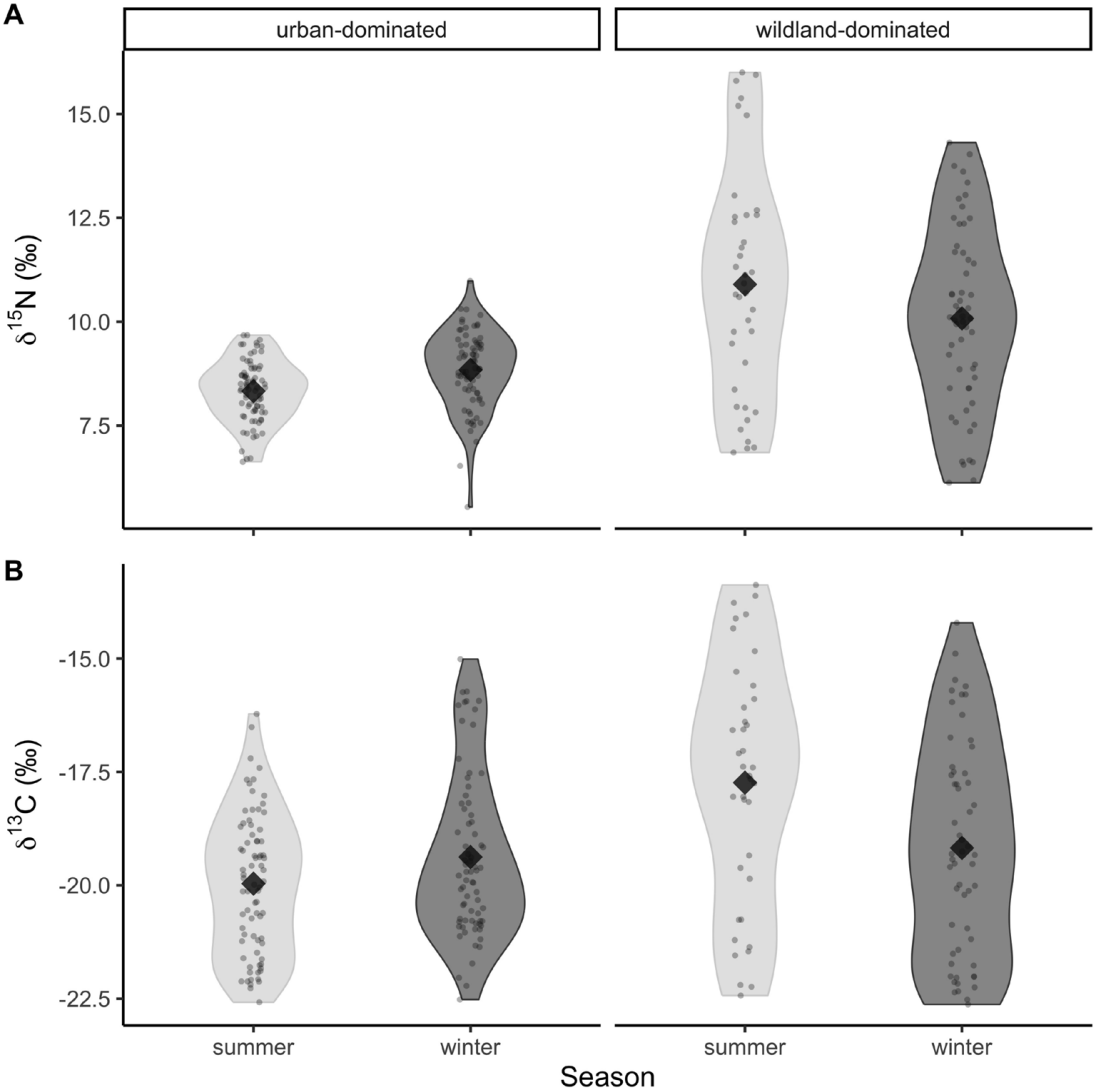
Violin plots with raw data points and means plotted as diamonds for (**A**) *δ* ^15^N and (**B**) *δ* ^13^C values. Data represent values in the summer and winter months, and caracals with urban‐dominated (*n* = 18) and wildland‐dominated (*n* = 10) home ranges on the Cape Peninsula, South Africa.

The LMMs for categorical variables of age class, sex, and type revealed that, when accounting for individual variation, males and females did not have significantly different *δ* ^15^N and *δ* ^13^C values (*p* > 0.05; see model estimates in Table 3). Subadult caracals had higher *δ* ^13^C than adults, although this was marginally significant (*β* = 1.25 ± 0.68, *p* = 0.07). There were no significant differences between age classes for *δ* ^15^N. Caracals with wildland‐dominated home ranges had higher *δ* ^15^N (mean = 10.4 ± 2.44) than those with urban‐dominated home ranges (mean = 8.58 ± 0.88; β = −1.51 ± 0.59, *p* < 0.05; Figure 3). There was seasonality in *δ* ^13^C, with values in winter (mean = −19.3 ± 2.05) being significantly lower than in summer (mean = −19.2 ± 2.24; *β* = −0.61 ± 0.2, *p* < 0.01; Figure 3). There was no significant difference between seasons for *δ* ^15^N values (*p* > 0.05; Figure 3).

**TABLE 3:** Model estimates for linear mixed models of *δ* ^15^N and *δ* ^13^C values in whisker segments sampled from caracals on the Cape Peninsula, South Africa. Top models for spatial and dietary models were selected from full models using AICc.

Model	Season and Demographics *δ* ^13^C		Season and Demographics *δ* ^15^N		Season and Spatial *δ* ^13^C		Season and Spatial *δ* ^15^N		Season and Dietary *δ* ^13^C		Season and Dietary *δ* ^15^N	
Predictors	Estimates	CI	Estimates	CI	Estimates	CI	Estimates	CI	Estimates	CI	Estimates	CI
Intercept	−18.99***	−20.25 to −17.73	10.02***	9.00 to 11.04	−18.91***	−19.63 to −18.20	9.79***	8.52 to 11.06	−19.21***	−20.06 to −18.35	8.57***	7.95 to 9.20
Age [subadult]	1.13	−0.26 to 2.52	0.52	−0.61 to 1.65								
Sex [male]	0.3	−1.16 to 1.75	0.02	−1.16 to 1.20								
Type [urban‐dominated]	−0.75	−2.17 to 0.67	−1.51*	−2.67 to −0.36								
Season [winter]	−0.61**	−1.02 to −0.20	0.01	−0.30 to 0.31	−0.63**	−1.04 to −0.22			−0.63**	−1.08 to −0.18	−0.11	−0.47 to 0.25
Wetlands							5.64	−0.66 to 11.94				
Coast distance							−0.38	−0.80 to 0.04				
Wetland biomass									2.09	−0.38 to 4.56	1.88*	0.23 to 3.52
Marine biomass											0.4	−1.36 to 2.15
Season [winter] × marine biomass											2.38*	0.44 to 4.33
**Random effects**												
σ^2^	1.13		0.64		1.13		0.64		1.2		0.59	
τ_00_	2.76 _caracal_id_		1.83 _caracal_id_		0.00 _type_		0.00 _type_		0.00 _type_		0.00 _type_	
					3.18 _caracal_id_		1.79 _caracal_id_		2.15 _caracal_id_		0.86 _caracal_id_	
ICC	0.71		0.74		0.74		0.74		0.64		0.59	
N	28 _caracal_id_		28 _caracal_id_		2 _type_		2 _type_		2 _type_		2 _type_	
					28 _caracal_id_		28 _caracal_id_		23 _caracal_id_		23 _caracal_id_	
Observations	244		244		244		244		202		202	
Marginal *R* ^2^/Conditional *R* ^2^	0.117/0.743		0.196/0.792		0.022/0.743		0.211/0.792		0.078/0.669		0.220/0.684	

* *p* < 0.05.

** *p* < 0.01.

*** *p* < 0.001.

### 3.2 Individual Specialisation

**FIGURE 4:**
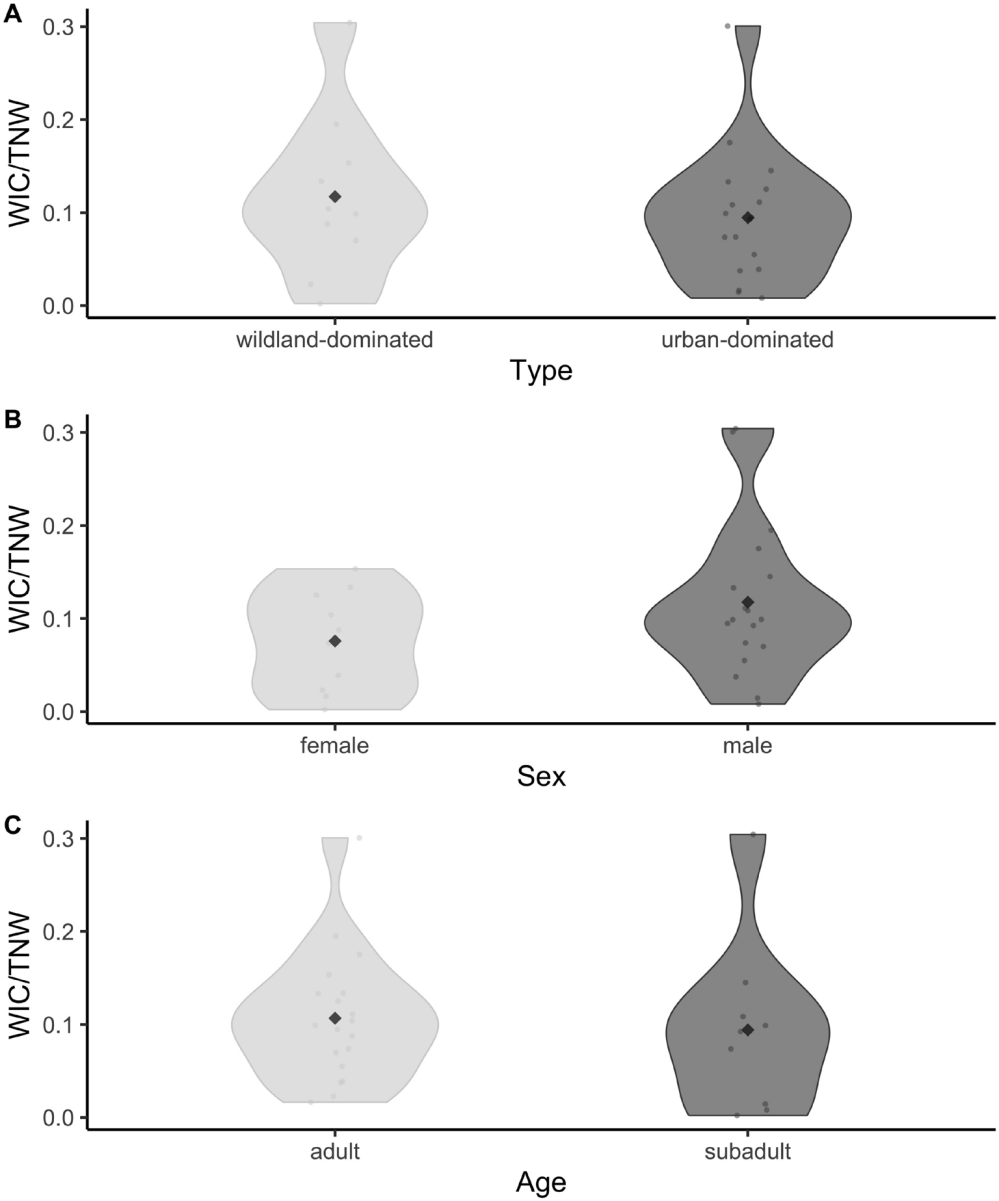
WIC/TNW ratios for caracals with (**A**) wildland‐dominated (*n* = 10) and urban‐dominated home ranges (*n* = 18) of the Cape Peninsula, South Africa, (**B**) female (*n* = 10) and male (*n* = 18) caracals, and (**C**) adult (*n* = 19) and subadult (*n* = 9) caracals. The WIC/TNW ratio is a measurement that represents the degree of individual diet specialization within a population. When the ratio approaches 1, all individuals utilize the full range of the population's niche.

Despite the high level of overlap between individuals (Figure S3), the WIC/TNW ratio (a measure of feeding specialisation) was low (< 0.35) and not significantly different between caracal groups (*p* > 0.05; Figure 4). The mean WIC/TNW was 0.095 ± 0.07 and 0.12 ± 0.09 for caracals with wildland‐ and urban‐dominated homes (Figure 4A), 0.08 ± 0.05 and 0.12 ± 0.08 for female and male individuals (Figure 4B), and 0.11 ± 0.07 and 0.09 ± 0.09 for adults and subadults, respectively (Figure 4C).

### 3.3 Isotopic Niche Space for Urban and Wildland Caracals

**FIGURE 5:**
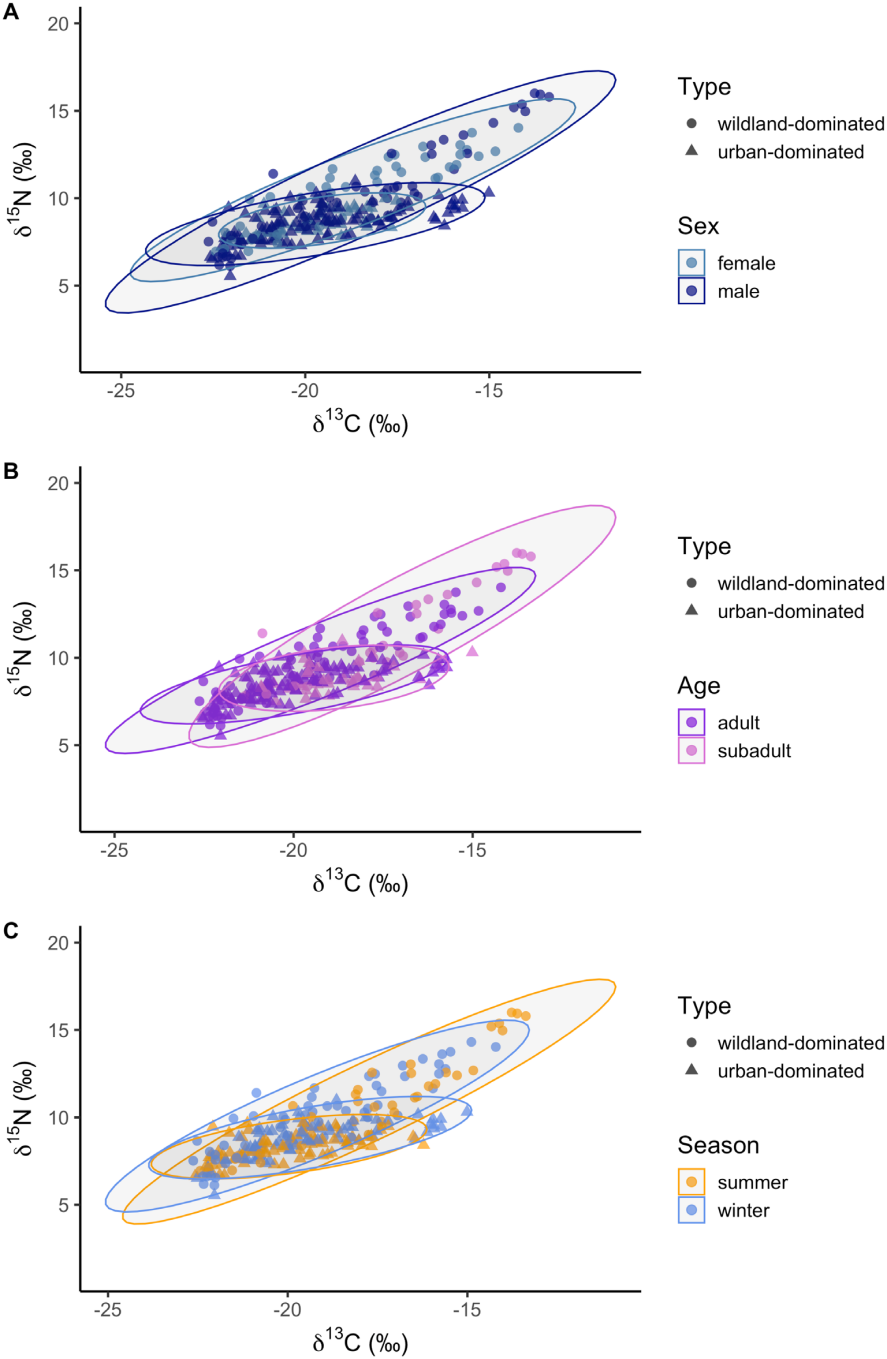
Bayesian ellipses of isotopic niche space for caracals with urban‐dominated and wildland‐dominated home ranges on the Cape Peninsula by (A) sex, (B) age and (C) season. Each data point represents the values for a single whisker segment.

**FIGURE 6:**
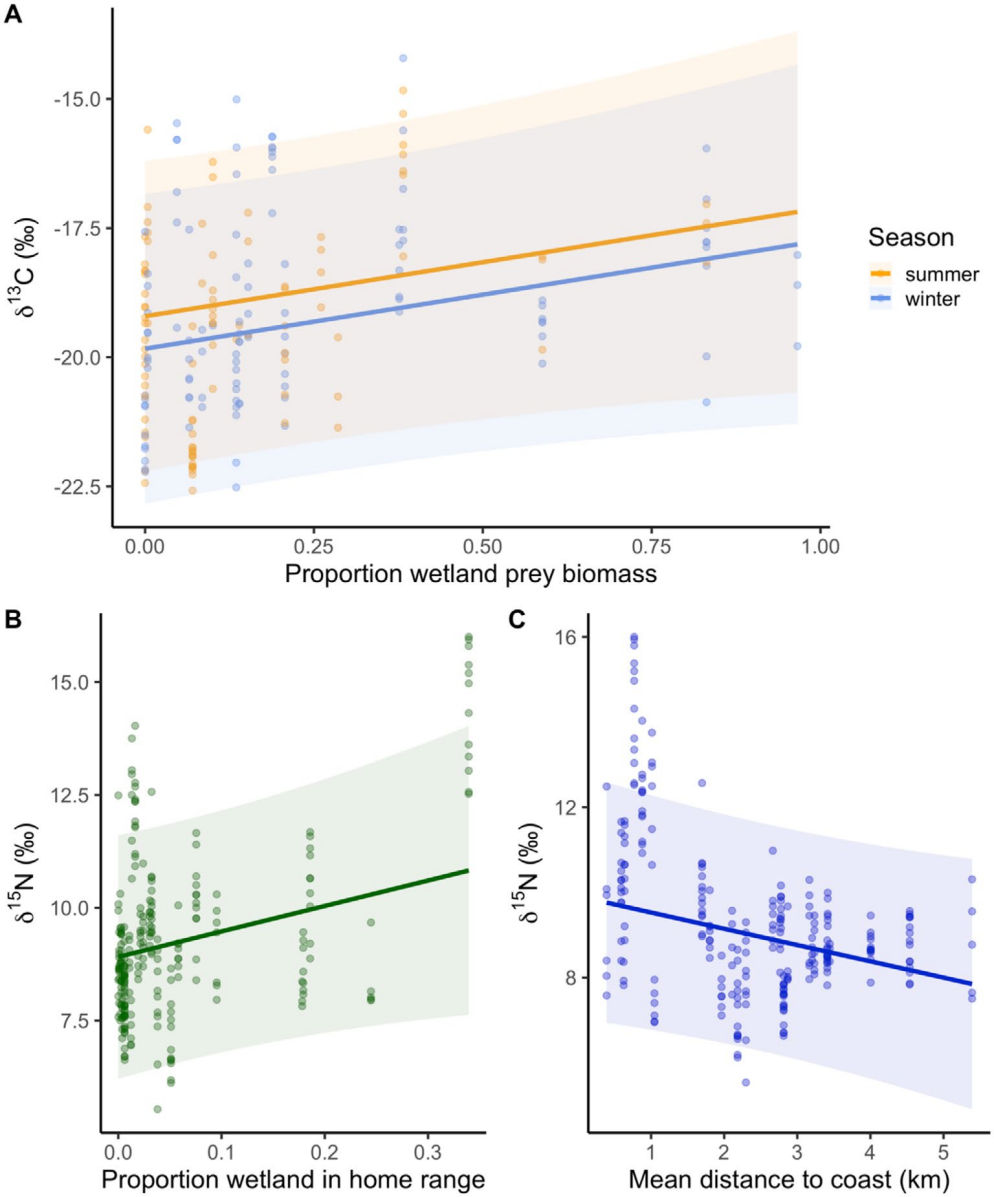
Prediction plots for linear mixed models of (A) *δ* ^13^C and proportion of wetland‐adapted prey in caracal diet (biomass) and *δ* ^15^N and (B) the proportion of wetland area in caracal home ranges and (C) the mean distance to coastline in home range. LMMs included the type of home range (urban‐ vs. wildland‐dominated) and caracal individual as a random effect.

The Bayesian ellipses revealed that males with wildland home ranges (TA = 30.82; SEAc = 9.22; Figure 5A) have larger isotopic niches than females in the corresponding area (TA = 18.66; SEAc = 6.68; Figure 6A), with 94.08% of male ellipses being larger than those of the females. Similarly, all urban male individuals demonstrated larger isotopic niches (TA = 22.57; SEAc = 4.29) than urban females (TA = 7.19; SEAc = 1.90; Figure 5A) with 100% of urban males' ellipses being larger. The comparison of posterior distributions showed that all (100%) of the female and male isotopic niches are smaller for those with urban home ranges than for those with wildland home ranges. This was supported by the Layman metrics, which showed that males have a larger range of both *δ* ^15^N and *δ* ^13^C values and larger total ellipse areas (Table S2). Adult individuals with wildland home ranges (TA = 24.01; SEAc = 6.72) and urban home ranges (TA = 19.27; SEAc = 3.71) had larger isotopic niches than the wildland (TA = 23.95; SEAc = 8.62) and urban (TA = 11.58; SEAc = 2.83; Figure 5B) subadults. The comparison of posterior distributions revealed that 91.65% of adult ellipses are larger than subadult ellipses for caracals with wildland home ranges, and 94.25% of ellipses for adults with urban home ranges are larger than those of urban subadults. Overall, caracals with urban home ranges had smaller isotopic niches (SEAc = 3.65) than individuals with wildland home ranges (SEAc = 8.10). For season, the comparison of posterior distributions showed that 84.88% of ellipses for caracals with wildland home ranges were larger in summer (TA = 21.18, SEAc = 8.27) than in winter (TA = 26.42, SEAc = 7.22; Figure 5C). In contrast, 94.25% of Bayesian ellipses for the caracals with urban home ranges were smaller in summer (TA = 12.42, SEAc = 3.02) compared to winter (TA = 18.60, SEAc = 4.04). All (100%) ellipses, in both summer and winter, were smaller for the individuals with urban‐ compared to the wildland‐dominated home ranges (Figure 5C).

### 3.4 The Effects of Spatiotemporal and Dietary Variables on *δ* ^15^N and *δ* ^13^C Signatures

According to model selection based on AICc, the top LMMs with spatiotemporal variables revealed a strong seasonal signal in *δ* ^13^C, where values in caracal whiskers were lower in winter (*β*_winter_ = −0.63 ± 0.21, *p* < 0.01), regardless of patterns within individual or between wildland or urban home ranges (random effects of caracal ID and type; see full model estimates in Table 3). In addition to this seasonal trend, the dietary LMM suggests that caracals feeding on more wetland‐adapted prey have enriched *δ* ^13^C signatures, although this is only marginally significant (*β* = 2.09 ± 1.26, *p* = 0.09; see Figure 6A). Terrestrial and arboreal‐adapted prey categories were collinear and thus removed from the top model (VIF > 3). There was no significant effect of season on *δ* ^15^N values, although the top spatial LMM revealed that caracals using more wetland areas (*β* = 5.64 ± 3.21, *p* = 0.08; Figure 6B) and areas closer to the coast (*β* = 0.38 ± 0.22, *p* = 0.07; Figure 6C) were more enriched, although these trends were also only marginally significant. Similar to *δ* ^13^C, the dietary models for *δ* ^15^N showed a positive association with wetland prey biomass (*β* = 1.88 ± 0.84, *p* < 0.05). The top diet LMM also included an interaction between season and the proportion of marine prey biomass. A higher proportion of marine prey in the diet was associated with enriched *δ* ^15^N values (β = 0.40 ± 0.90, *p* = 0.66), but this trend was stronger in winter (*β* = 2.38 ± 0.99, *p* < 0.05). Demographic group and the proportion of urban area in the caracal home range were not in any of the top spatial or dietary LMMs with random effects of type and caracal ID (Table 3).

## 4 Discussion

We used a stable isotope approach to explore individual dietary flexibility in an adaptable carnivore utilising a rapidly urbanising landscape, assessing the influence of seasonality and exposure to urban and coastal edges on feeding ecology. Our results reveal significant roles for demographics and multiple spatiotemporal variables in the trophic ecology and isotopic niche patterns of Cape Town's caracal population. Using home range estimates and SIA of *δ* ^13^C and *δ* ^15^N isotope ratios from caracal whisker samples, individual age and sex, as well as season and habitat use, were found to be important attributes of the *δ* ^13^C and *δ* ^15^N isoscape of individual caracals. Further, levels of exposure to urbanisation appear to be an important contributor to the isotope ecology of this ecologically flexible species. Together, these findings suggest that demographics and urbanisation may influence inter‐ and intra‐specific niche variation, potentially leading to altered competition between individuals and predator–prey relationships within this urbanising system. These results contribute to a more nuanced understanding of the importance of the highly flexible trophic ecology of carnivores in increasingly human‐impacted landscapes.

### 4.1 Sex and Age Effects on Isotopic Niche

There was limited age structuring in *δ* ^13^C values, with subadults having marginally enriched signatures compared to adults. This may be the result of differences in habitat selection preferences, which differ between adult and subadult caracals on the Cape Peninsula (Leighton et al. 2022b; Serieys et al. 2023). In contrast to previous isotope analyses of Cape Town's caracals based on individual fur samples (Leighton et al. 2023), isotopic niche analysis of whiskers revealed narrower niches for subadults using both wildland‐ and urban‐dominated areas. Using multiple whisker segments for SIA may provide finer scale insight into the isotopic signatures of individuals than using single samples of fur alone, potentially accounting for the different pattern observed (Rogers et al. 2020). Alternatively, this may be the result of competitive exclusion of subadults by adult caracals from valuable resources, thus limiting their niche space (Leighton et al. 2022b; Serieys et al. 2023). Similar exclusion effects have been reported in numerous carnivore species within transformed landscapes, including medium‐sized felids, such as bobcats ( *Lynx rufus* ; Riley et al. 2003) and Iberian lynx ( *Lynx pardinus* ; Palomares et al. 2000). Competitive exclusion might be intensified in rapidly fragmenting human‐modified landscapes, making it more difficult for subadults to establish home ranges while pushed into sub‐optimal habitats by dominant adults (Riley et al. 2003). Similarly, while *δ* ^13^C and *δ* ^15^N values did not differ significantly between male and female individuals, males had a larger isotopic niche than females using both urban‐ and wildland‐dominated areas. These findings may be the result of greater mobility, as males typically have larger home ranges (Leighton et al. 2022b; Table S1), and may therefore exploit a greater diversity of prey items across different trophic levels (Leighton et al. 2020).

### 4.2 Individual Caracals Demonstrate Remarkable Dietary Flexibility, From Generalist to Specialist

Individual specialisation has important ecological, evolutionary, and conservation implications, as it can affect population stability and resilience, intraspecific competition, and the capacity of populations to diversify and speciate rapidly (Bolnick et al. 2003). As with urban‐adapted populations of red foxes (Scholz et al. 2020) and coyotes (Newsome et al. 2015a), caracals show high levels of individual specialisation within a generalist population (low WIC/TNW ratios; Figure 4). This suggests that urban populations of generalist carnivores may often include specialist individuals (Bolnick et al. 2003, 2007; Newsome et al. 2015a; Scholz et al. 2020). The lack of significant differences between the level of specialisation among caracal grouping variables used in our analyses suggests that individual specialisation is not necessarily influenced by urbanisation or demographics, but rather that Cape Town's caracals occupy multiple diverse niches within the population's total potential niche range. Differences in diet composition and dietary specialisation have been observed across various taxa and habitats, and such variation at the individual level is increasingly recognised as an important component of diversity in trophic interactions (Bolnick et al. 2002, 2003). While individual specialisation can comprise the majority of the population's niche width, the degree of individual specialisation varies widely among species and populations, reflecting a diversity of physiological, behavioural, and ecological mechanisms that can generate intrapopulation variation (Bolnick et al. 2003).

### 4.3 Spatiotemporal Variation in Foraging Behaviour Influences Niche Space

Smaller isotopic niches characterised individuals using more urban areas of the Cape Peninsula than those using more wildland areas. This pattern is maintained when accounting for age and sex within the Peninsula caracals and suggests a degree of habitat‐specific foraging. This reduced niche width may influence inter‐ and intra‐specific niche variation, leading to greater overlap and therefore competition within the system (Layman et al. 2007). The larger niches of individuals with wildland‐dominated home ranges may represent niche expansion of coastal foraging individuals with access to marine dietary resources, such as coastal seabirds (Leighton et al. 2024). Previous isotope analysis of fur samples of both monitored and opportunistically sampled individuals suggests that marine resources are important in the diet of Cape Town's caracals with wildland‐dominated home ranges (Leighton et al. 2024), and further reveals their opportunistic dietary flexibility. Within the study area, caracals are known to hunt seabirds (e.g., Cape cormorants ( *Phalacrocorax capensis* ), gulls ( *Chroicocephalus hartlaubii* and *Larus dominicanus* ), and African penguins ( *Spheniscus demersus* ) (Leighton et al. 2020)). Other terrestrial predators also utilise marine and freshwater prey species as fundamental sources of protein during specific periods of the year (e.g., wolves ( *Canis lupus* ) and black bears ( *Ursus americanus* ); Bonin et al. 2023). Similarly, we find *δ* ^15^N enrichment, which is associated with increased consumption of marine resources (Peterson and Fry 1987; Darimont et al. 2009), particularly in winter in the Cape region, suggesting that marine prey may be particularly important resources for individual caracals during the colder, wetter months.

Across the Cape Peninsula, both *δ* ^13^C and *δ* ^15^N values were also positively associated with increased foraging of caracal on wetland‐adapted prey, which from previous scat analysis are mainly Egyptian geese ( *Alopochen aegyptiaca* ), African sacred ibises ( *Threskiornis aethiopicus* ) and vlei rats ( *Otomys irroratus* ). Further, the use of wetland areas in caracal home ranges was weakly associated with enriched *δ* ^15^N. Highly eutrophic wetlands near developed areas are often associated with enriched isotope signatures (Inglett and Reddy 2006; Zheng et al. 2019). Importantly, both marine and wetland foraging in Cape Town's caracal is also associated with exposure to environmental pollutants, such as DDT, PCBs and metals, revealing some of the cryptic costs associated with foraging near cities (Leighton et al. 2022a; Parker et al. 2023).

Differences in niche size for species able to successfully exploit urban resources may also reflect niche collapse in urban habitats where increased hunting efficiency may favour the targeting of hyperabundant low trophic level, and arguably low value, prey species associated with urban areas (Layman et al. 2007; Leighton et al. 2023). Seasonal changes in niches may also be impacted by the degree of urbanisation (Shochat 2004; Vardi and Berger‐Tal 2022), with niche contraction in summer for caracals using urban‐dominated areas and niche expansion in summer for caracals using wildland‐dominated areas. Despite niches being relatively smaller for caracals using more urban areas, their contraction during the summer months is more significant, along with significant increases in *δ* ^13^C values during summer across the Cape Peninsula. This may reflect a dietary focus on anthropogenic prey (i.e., exotic or human‐associated species) with stronger C_4_ signals (Jahren et al. 2008; Gámez et al. 2022) that do well in the hot, dry summer months because of access to watered and fertilized gardens and urban greenbelt areas (Newsome et al. 2015b; Suri et al. 2017). Together these findings suggest that reliance on anthropogenic subsidies around cities may vary temporally, with implications for ecosystems, communities and trophic interactions through altering processes like competition, predator–prey interactions, as well as nutrient transfer within and between biotopes (Oro et al. 2013; Newsome et al. 2015b).

### 4.4 Limitations of Whisker Isotope Analysis for Smaller Carnivores

Unlike studies on larger marine and terrestrial carnivores (e.g., Kernaléguen et al. 2012; Mutirwara et al. 2018; Voigt et al. 2018; McHuron et al. 2019), whisker sampling for caracals in our study system provides a relatively small window into diet patterns over time. As a medium‐sized wild cat with shorter, lighter vibrissae, whiskers produced, on average, six usable segments, representing only 1–2 months of growth. The distal ends of the whisker also had to be combined to achieve the mass required for SIA, which may have masked temporal trends in older sections of the whisker. The limited availability of species‐specific growth rates for whiskers also limits the accuracy of dating samples and thus assessing seasonal trends in species foraging ecology. Despite the challenges of analysing whisker material, this study highlights its value in providing novel insight into the short‐term intra‐individual variation in the trophic ecology of caracal in a rapidly urbanising landscape.

## 5 Conclusion

This study contributes to our understanding of behaviourally flexible urban wildlife globally, and particularly in the generally poorly studied Global South. Understanding the resource requirements, dietary niches, and impacts of urbanisation on the foraging ecology of urban carnivores is fundamental for ensuring their persistence in rapidly urbanising systems. SIA results suggest that Cape Town's caracals are highly flexible and able to exploit a diverse array of resources, both on the urban and coastal edges of a peninsula system isolated by the city's extensive urban development. SIA of whisker segments reveals interesting seasonal patterns, suggesting the use of marine and anthropogenic resources likely varies temporally. Here, we add to the growing evidence that ecologically generalist urban wildlife populations often comprise specialist individuals who demonstrate a high degree of variation across a range of urbanisation. Our results highlight the impact urbanisation has on carnivore ecology and ecosystem processes, with important potential consequences for competition and predator–prey dynamics. These findings inform our understanding of urban adapters and how intensifying urbanisation may impact wildlife persistence in rapidly developing areas.

## Author Contributions

**Gabriella R. M. Leighton:** conceptualization (equal), data curation (lead), formal analysis (equal), visualization (equal), writing – original draft (equal). **Anna R. Brooke:** conceptualization (equal), formal analysis (equal), writing – original draft (equal). **P. William Froneman:** conceptualization (equal), resources (equal), supervision (equal), writing – review and editing (equal). **Laurel E. K. Serieys:** conceptualization (equal), resources (equal), writing – review and editing (equal). **Jacqueline M. Bishop:** conceptualization (equal), resources (equal), supervision (equal), writing – review and editing (equal).

## Conflicts of Interest

The authors declare no conflicts of interest.

## Supporting information


**Appendix S1.** Supporting Information.

## Data Availability

Data will be available from the Dryad Digital Repository (https://doi.org/10.5061/dryad.9ghx3fft8). Reviewer sharing link: http://datadryad.org/stash/share/jJ7ESFeKIyZpCPrRAIZIg2oP4s97ZpBY‐xxdxilTTFw.

## References

[ece371154-bib-0001] Alberti, M. 2015. “Eco‐Evolutionary Dynamics in an Urbanizing Planet.” Trends in Ecology & Evolution 30: 114–126. 10.1016/j.tree.2014.11.007. 25498964

[ece371154-bib-0002] Allsopp, N. , J. F. Colville , and G. A. Verboom . 2014. Fynbos: Ecology, Evolution, and Conservation of a Megadiverse Region. Oxford University Press.

[ece371154-bib-0003] Anderson, P. M. L. L. , and P. J. G. O'Farrell . 2012. “An Ecological View of the History of the City of Cape Town.” Ecology and Society 17, no. 3: art28. 10.5751/ES-04970-170328.

[ece371154-bib-0047] Atiqah, N. , Z. Akbar , S. Syafrinna , N. Ubaidah , and N. Y. Foo . 2015. “Comparison of the Ranging Behavior of *Scotophilus kuhlii* (Lesser Asiatic Yellow Bat) in Agricultural and Urban Landscape.” AIP Conference Proceedings 1678, no. 1: 020026. 10.1063/1.4931211.

[ece371154-bib-0004] Attard, M. R. G. G. , A. Lewis , S. Wroe , M. R. G. Attard , C. Hughes , and T. L. Rogers . 2021. “Whisker Growth in Tasmanian Devils (*Sarcophilus harrisii*) and Applications for Stable Isotope Studies.” Ecosphere 12, no. 11: e03846. 10.1002/ecs2.3846.

[ece371154-bib-0005] Avenant, N. L. , and J. A. J. Nel . 2002. “Among Habitat Variation in Prey Availability and Use by Caracal *Felis caracal* .” Mammalian Biology 67: 18–33. 10.1078/1616-5047-00002.

[ece371154-bib-0006] Bartoń, K. 2020. MuMIn: Multi‐Model Inference. R Package Version 1.43.17.

[ece371154-bib-0007] Bateman, P. W. , and P. A. Fleming . 2012. “Big City Life: Carnivores in Urban Environments.” Journal of Zoology 287: 1–23. 10.1111/j.1469-7998.2011.00887.x.

[ece371154-bib-0008] Ben‐David, M. , and E. A. Flaherty . 2012. “Stable Isotopes in Mammalian Research: A Beginner's Guide.” Journal of Mammalogy 93: 312–328. 10.1644/11-MAMM-S-166.1.

[ece371154-bib-0009] Birnie‐Gauvin, K. , K. S. Peiman , A. J. Gallagher , R. de Bruijn , and S. J. Cooke . 2016. “Sublethal Consequences of Urban Life for Wild Vertebrates.” Environmental Reviews 24, no. 4: 416–425. 10.1139/ER-2016-0029.

[ece371154-bib-0010] Bligh, E. G. , and W. J. Dyer . 1959. “A Rapid Method of Total Lipid Extraction and Purification.” Canadian Journal of Biochemistry and Physiology 37: 911–917. 10.1139/o59-099. 13671378

[ece371154-bib-0011] Bókony, V. , A. Kulcsár , Z. Tóth , and A. Liker . 2012. “Personality Traits and Behavioral Syndromes in Differently Urbanized Populations of House Sparrows ( *Passer domesticus* ).” PLoS One 7: e36639. 10.1371/journal.pone.0036639. 22574204 PMC3344926

[ece371154-bib-0012] Bolnick, D. I. , P. Amarasekare , M. S. Araújo , et al. 2011. “Why Intraspecific Trait Variation Matters in Community Ecology.” Trends in Ecology & Evolution 26: 183–192. 21367482 10.1016/j.tree.2011.01.009 PMC3088364

[ece371154-bib-0013] Bolnick, D. I. , R. Svanbäck , M. S. Araújo , and L. Persson . 2007. “Comparative Support for the Niche Variation Hypothesis That More Generalized Populations Also Are More Heterogeneous.” Proceedings of the National Academy of Sciences of the United States of America 104: 10075–10079. 10.1073/pnas.0703743104. 17537912 PMC1891261

[ece371154-bib-0014] Bolnick, D. I. , R. Svanbäck , J. A. Fordyce , et al. 2003. “The Ecology of Individuals: Incidence and Implications of Individual Specialization.” American Naturalist 161: 1–28. 10.1086/343878 12650459

[ece371154-bib-0015] Bolnick, D. I. , L. H. Yang , J. A. Fordyce , J. M. Davis , and R. Svanbäck . 2002. “Measuring Individual‐Level Resource Specialization.” Ecology 83: 2936–2941. 10.1890/0012-9658(2002)083[2936:MILRS]2.0.CO;2.

[ece371154-bib-0016] Bonin, M. , C. Dussault , J. Taillon , J. Pisapio , N. Lecomte , and S. D. Côté . 2023. “Diet Flexibility of Wolves and Black Bears in the Range of Migratory Caribou.” Journal of Mammalogy 104: 252–264. 10.1093/jmammal/gyad002.

[ece371154-bib-0017] Burnham, K. P. , and D. R. Anderson . 2002. “Avoiding Pitfalls When Using Information‐Theoretic Methods.” Journal of Wildlife Management 66: 912–918.

[ece371154-bib-0018] Cecchetti, M. , S. L. Crowley , C. E. D. Goodwin , et al. 2021. “Contributions of Wild and Provisioned Foods to the Diets of Domestic Cats That Depredate Wild Animals.” Ecosphere 12, no. 9: e03737. 10.1002/ecs2.3737.

[ece371154-bib-0019] Chakrabarti, S. , Y. V. Jhala , S. Dutta , Q. Qureshi , R. F. Kadivar , and V. J. Rana . 2016. “Adding Constraints to Predation Through Allometric Relation of Scats to Consumption.” Journal of Animal Ecology 85: 660–670. 10.1111/1365-2656.12508. 26931378

[ece371154-bib-0020] Cowling, R. M. , I. A. W. MacDonald , and M. T. Simmons . 1996. “The Cape Peninsula, South Africa: Physiographical, Biological and Historical Background to an Extraordinary Hot‐Spot of Biodiversity.” Biodiversity and Conservation 5: 527–550. 10.1007/BF00137608.

[ece371154-bib-0021] Darimont, C. T. , P. C. Paquet , and T. E. Reimchen . 2009. “Landscape Heterogeneity and Marine Subsidy Generate Extensive Intrapopulation Niche Diversity in a Large Terrestrial Vertebrate.” Journal of Animal Ecology 78: 126–133. 10.1111/j.1365-2656.2008.01473.x. 19120600

[ece371154-bib-0022] Ditchkoff, S. S. , S. T. Saalfeld , and C. J. Gibson . 2006. “Animal Behavior in Urban Ecosystems: Modifications due to Human‐Induced Stress.” Urban Ecosystem 9: 5–12. 10.1007/s11252-006-3262-3.

[ece371154-bib-0023] Fleming, P. A. , and P. W. Bateman . 2018. “Novel Predation Opportunities in Anthropogenic Landscapes.” Animal Behaviour 138: 145–155. 10.1016/j.anbehav.2018.02.011.

[ece371154-bib-0024] Funston, P. J. , M. G. L. Mills , H. C. Biggs , and P. R. K. Richardson . 1998. “Hunting by Male Lions: Ecological Influences and Socioecological Implications.” Animal Behaviour 56: 1333–1345. 9933529 10.1006/anbe.1998.0884

[ece371154-bib-0025] Gámez, S. , A. Potts , K. L. Mills , et al. 2022. “Downtown Diet: A Global Meta‐Analysis of Increased Urbanization on the Diets of Vertebrate Predators.” Proceedings of the Royal Society B: Biological Sciences 289: 20212487. 10.1098/rspb.2021.2487. PMC8889190 35232241

[ece371154-bib-0026] Getz, W. M. , S. Fortmann‐Roe , P. C. Cross , A. J. Lyons , S. J. Ryan , and C. C. Wilmers . 2007. “LoCoH: Nonparameteric Kernel Methods for Constructing Home Ranges and Utilization Distributions.” PLoS One 2, no. 2: 1–11. 10.1371/journal.pone.0000207. PMC1797616 17299587

[ece371154-bib-0027] Hawkins, D. M. 2004. “The Problem of Overfitting.” Journal of Chemical Information and Computer Sciences 44: 1–12. 14741005 10.1021/ci0342472

[ece371154-bib-0028] Holekamp, K. E. , E. M. Swanson , and P. E. Van Meter . 2013. “Developmental Constraints on Behavioural Flexibility.” Philosophical Transactions of the Royal Society, B: Biological Sciences 368, no. 1618: 20120350. 10.1098/RSTB.2012.0350. 23569298 PMC3638453

[ece371154-bib-0029] Inglett, P. W. , and K. R. Reddy . 2006. “Investigating the Use of Macrophyte Stable C and N Isotopic Ratios as Indicators of Wetland Eutrophication: Patterns in the P‐Affected Everglades.” Limnology and Oceanography 51: 2380–2387. 10.4319/lo.2006.51.5.2380.

[ece371154-bib-0030] Jahren, A. H. , R. A. Kraft , and S. M. Stanley . 2008. “Carbon and Nitrogen Stable Isotopes in Fast Food: Signatures of Corn and Confinement.” National Academy of Sciences 105, no. 46: 17855–17860. 10.1073/pnas.0809870105 PMC2582047 19001276

[ece371154-bib-0031] Kernaléguen, L. , B. Cazelles , J. P. Y. Y. Arnould , et al. 2012. “Long‐Term Species, Sexual and Individual Variations in Foraging Strategies of Fur Seals Revealed by Stable Isotopes in Whiskers.” PLoS One 7: e32916. 10.1371/JOURNAL.PONE.0032916. 22431988 PMC3303799

[ece371154-bib-0032] Layman, C. A. , J. P. Quattrochi , C. M. Peyer , and J. E. Allgeier . 2007. “Niche Width Collapse in a Resilient Top Predator Following Ecosystem Fragmentation.” Ecology Letters 10: 937–944. 10.1111/J.1461-0248.2007.01087.X. 17845294 PMC2040226

[ece371154-bib-0033] Lee‐Thorp, J. A. , J. C. Sealy , and N. J. van der Merwe . 1989. “Stable Carbon Isotope Ratio Differences Between Bone Collagen and Bone Apatite, and Their Relationship to Diet.” Journal of Archaeological Science 16: 585–599. 10.1016/0305-4403(89)90024-1.

[ece371154-bib-0034] Leighton, G. R. M. , J. M. Bishop , P. R. Camarero , R. Mateo , M. J. O'Riain , and L. E. K. Serieys . 2022a. “Poisoned Chalice: Use of Transformed Landscapes Associated With Increased Persistent Organic Pollutant Concentrations and Potential Immune Effects for an Adaptable Carnivore.” Science of the Total Environment 822: 153581. 10.1016/j.scitotenv.2022.153581. 35104517

[ece371154-bib-0035] Leighton, G. R. M. , J. M. Bishop , M. J. O'Riain , et al. 2020. “An Integrated Dietary Assessment Increases Feeding Event Detection in an Urban Carnivore.” Urban Ecosystem 23, no. 3: 569–583. 10.1007/s11252-020-00946-y.

[ece371154-bib-0036] Leighton, G. R. M. , P. W. Froneman , L. E. K. Serieys , and J. M. Bishop . 2024. “Sustained Use of Marine Subsidies Promotes Niche Expansion in a Wild Felid.” Science of the Total Environment 914: 169912. 10.1016/j.scitotenv.2024.169912. 38184259

[ece371154-bib-0037] Leighton, G. R. M. , W. Froneman , L. E. K. Serieys , and J. M. Bishop . 2023. “Trophic Downgrading of an Adaptable Carnivore in an Urbanising Landscape.” Scientific Reports 13: 1–12. 10.1038/s41598-023-48868-x. 38062237 PMC10703923

[ece371154-bib-0038] Leighton, G. R. M. M. , J. M. Bishop , J. Merondun , et al. 2022b. “Hiding in Plain Sight: Risk Mitigation by a Cryptic Carnivore Foraging at the Urban Edge.” Animal Conservation 25, no. 2: 244–258. 10.1111/acv.12732.

[ece371154-bib-0039] Lowry, H. , A. Lill , and B. B. M. Wong . 2013. “Behavioural Responses of Wildlife to Urban Environments.” Biological Reviews 88: 537–549. 10.1111/BRV.12012. 23279382

[ece371154-bib-0040] Lyons, A. J. , W. C. Turner , and W. M. Getz . 2013. “Home Range Plus: A Space‐Time Characterization of Movement Over Real Landscapes.” Movement Ecology 1: 2. 10.1186/2051-3933-1-2. 25709816 PMC4337754

[ece371154-bib-0041] Mazza, V. , M. Dammhahn , E. Lösche , and J. A. Eccard . 2020. “Small Mammals in the Big City: Behavioural Adjustments of Non‐Commensal Rodents to Urban Environments.” Global Change Biology 26: 6326–6337. 10.1111/gcb.15304. 32767603

[ece371154-bib-0042] McHuron, E. A. , R. R. Holser , and D. P. Costa . 2019. “What's in a Whisker? Disentangling Ecological and Physiological Isotopic Signals.” Rapid Communications in Mass Spectrometry 33: 57–66. 10.1002/rcm.8312. 30334287

[ece371154-bib-0043] McHuron, E. A. , S. M. Walcott , J. Zeligs , S. Skrovan , D. P. Costa , and C. Reichmuth . 2016. “Whisker Growth Dynamics in Two North Pacific Pinnipeds: Implications for Determining Foraging Ecology From Stable Isotope Analysis. int‐res.com.” Marine Ecology Progress Series 554: 213–224. 10.3354/meps11793.

[ece371154-bib-0044] McKinney, M. L. 2002. “Urbanization, Biodiversity and Conservation.” Bioscience 52: 883–890.

[ece371154-bib-0045] Moss, W. E. , M. W. Alldredge , K. A. Logan , and J. N. Pauli . 2016. “Human Expansion Precipitates Niche Expansion for an Opportunistic Apex Predator (*Puma concolor*).” Scientific Reports 6: 1–5. 10.1038/srep39639. 28008961 PMC5180354

[ece371154-bib-0046] Mutirwara, R. , F. G. T. T. Radloff , and D. Codron . 2018. “Growth Rate and Stable Carbon and Nitrogen Isotope Trophic Discrimination Factors of Lion and Leopard Whiskers.” Rapid Communications in Mass Spectrometry 32: 33–47. 10.1002/rcm.8003. 28971533

[ece371154-bib-0048] Newsome, S. D. , H. M. Garbe , E. C. Wilson , and S. D. Gehrt . 2015a. “Individual Variation in Anthropogenic Resource Use in an Urban Carnivore.” Oecologia 178: 115–128. 10.1007/S00442-014-3205-2. 25669449

[ece371154-bib-0049] Newsome, T. M. , J. A. Dellinger , C. R. Pavey , et al. 2015b. “The Ecological Effects of Providing Resource Subsidies to Predators.” Global Ecology and Biogeography 24: 1–11. 10.1111/geb.12236.

[ece371154-bib-0050] Oro, D. , M. Genovart , G. Tavecchia , M. S. Fowler , and A. Martínez‐Abraín . 2013. “Ecological and Evolutionary Implications of Food Subsidies From Humans.” Ecology Letters 16: 1501–1514. 10.1111/ELE.12187. 24134225

[ece371154-bib-0051] Palomares, F. , M. Delibes , P. Ferreras , J. M. Fedriani , J. Calzada , and E. Revilla . 2000. “Iberian Lynx in a Fragmented Landscape: Predispersal, Dispersal, and Postdispersal Habitats.” Conservation Biology 14: 809–818. 10.1046/j.1523-1739.2000.98539.x.

[ece371154-bib-0052] Parchizadeh, J. , S. L. Schooler , M. A. Adibi , et al. 2023. “A Review of Caracal and Jungle Cat Diets Across Their Geographical Ranges During 1842–2021.” Ecology and Evolution 13, no. 5: e10130. 10.1002/ece3.10130. 37250441 PMC10212689

[ece371154-bib-0053] Parker, K. H. , J. M. Bishop , L. E. K. Serieys , R. Mateo , P. R. Camarero , and G. R. M. Leighton . 2023. “A Heavy Burden: Metal Exposure Across the Land‐Ocean Continuum in an Adaptable Carnivore.” SSRN Electronic Journal 327: 121585. 10.2139/ssrn.4354807. 37040831

[ece371154-bib-0054] Peterson, B. J. , and B. Fry . 1987. “Stable Isotopes in Ecosystem Studies.” Annual Review of Ecology and Systematics 18: 293–320. 10.1146/annurev.es.18.110187.001453.

[ece371154-bib-0055] Pollard, M. D. , and E. E. Puckett . 2021. “Evolution of Degrees of Carnivory and Dietary Specialization Across Mammalia and Their Effects on Speciation.” bioRxiv. 10.1101/2021.09.15.460515.

[ece371154-bib-0056] QGIS Development Team . 2024. QGIS Geographic Information System Version 3.34.9‐Prizren. QGIS Association.

[ece371154-bib-0057] R Core Team, Team RC . 2024. R: A Language and Environment for Statistical Computing. https://www.r‐project.org/.

[ece371154-bib-0058] Riley, S. P. D. , J. A. Sikich , and J. F. Benson . 2021. “Big Cats in the Big City: Spatial Ecology of Mountain Lions in Greater Los Angeles.” Journal of Wildlife Management 85: 1527–1542. 10.1002/JWMG.22127.

[ece371154-bib-0059] Riley, S. P. D. D. , R. M. Sauvajot , T. K. Fuller , et al. 2003. “Effects of Urbanization and Habitat Fragmentation on Bobcats and Coyotes in Southern California.” Conservation Biology 17, no. 2: 566–576. 10.1046/j.1523-1739.2003.01458.x.

[ece371154-bib-0060] Ritzel, K. , and T. Gallo . 2020. “Behavior Change in Urban Mammals: A Systematic Review.” Frontiers in Ecology and Evolution 8: 1–11. 10.3389/fevo.2020.576665.

[ece371154-bib-0061] Rogers, M. C. , G. V. Hilderbrand , D. D. Gustine , et al. 2020. “Splitting Hairs: Dietary Niche Breadth Modelling Using Stable Isotope Analysis of a Sequentially Grown Tissue.” Isotopes in Environmental and Health Studies 56: 358–369. 10.1080/10256016.2020.1787404. 32631088

[ece371154-bib-0062] Santini, L. , M. González‐Suárez , D. Russo , A. Gonzalez‐Voyer , A. von Hardenberg , and L. Ancillotto . 2019. “One Strategy Does Not Fit all: Determinants of Urban Adaptation in Mammals.” Ecology Letters 22: 365–376. 10.1111/ele.13199. 30575254 PMC7379640

[ece371154-bib-0063] Scholz, C. , J. Firozpoor , S. Kramer‐Schadt , et al. 2020. “Individual Dietary Specialization in a Generalist Predator: A Stable Isotope Analysis of Urban and Rural Red Foxes.” Ecology and Evolution 10: 8855–8870. 10.1002/ece3.6584. 32884662 PMC7452770

[ece371154-bib-0064] Schroeder, M. A. , L. A. Robb , and C. Braun . 2005. “Criteria for Gender and Age.” In Techniques for Wildlife Investigations and Management, edited by N. J. Silvy , 303–338. Wildlife Society.

[ece371154-bib-0065] Serieys, L. E. K. , and J. M. Bishop . 2024. “Data from: Study Caracal Movement Ecology Study in Cape Town, South Africa.” Movebank Data Repository. 10.5441/001/1.317.

[ece371154-bib-0066] Serieys, L. E. K. , J. M. Bishop , M. S. Rogan , et al. 2023. “Anthropogenic Activities and Age Class Mediate Carnivore Habitat Selection in a Human‐Dominated Landscape.” iScience 26, no. 7: 1–27. 10.1016/j.isci.2023.107050. PMC10391726 37534145

[ece371154-bib-0067] Shochat, E. 2004. “Credit or Debit? Resource Input Changes Population Dynamics of City‐Slicker Birds.” Oikos 106: 622–626.

[ece371154-bib-0068] Sih, A. , M. C. O. Ferrari , and D. J. Harris . 2011. “Evolution and Behavioural Responses to Human‐Induced Rapid Environmental Change.” Evolutionary Applications 4: 367–387. 10.1111/j.1752-4571.2010.00166.x. 25567979 PMC3352552

[ece371154-bib-0069] Suraci, J. P. , K. M. Gaynor , M. L. Allen , et al. 2021. “Disturbance Type and Species Life History Predict Mammal Responses to Humans.” Global Change Biology 27, no. 16: 1–39. 10.1111/gcb.15650. 33887083

[ece371154-bib-0070] Suri, J. , P. Sumasgutner , É. Hellard , A. Koeslag , and A. Amar . 2017. “Stability in Prey Abundance May Buffer Black Sparrowhawks *Accipiter melanoleucus* From Health Impacts of Urbanization.” Ibis 159, no. 1: 38–54. 10.1111/ibi.12422.

[ece371154-bib-0071] Tredennick, A. T. , G. Hooker , S. P. Ellner , and P. B. Adler . 2021. “A Practical Guide to Selecting Models for Exploration, Inference, and Prediction in Ecology.” Ecology 102: e03336. 10.1002/ecy.3336. 33710619 PMC8187274

[ece371154-bib-0072] Vardi, R. , and O. Berger‐Tal . 2022. “Environmental Variability as a Predictor of Behavioral Flexibility in Urban Environments.” Behavioral Ecology 33: 573–581. 10.1093/BEHECO/ARAC002.

[ece371154-bib-0073] Venter, O. , E. W. Sanderson , and A. Magrach . 2018. Last of the Wild Project, Version 3 (LWP‐3): 2009 Human Footprint, 2018 Release.

[ece371154-bib-0074] Venter, O. , E. W. Sanderson , A. Magrach , et al. 2016. “Sixteen Years of Change in the Global Terrestrial Human Footprint and Implications for Biodiversity Conservation.” Nature Communications 7: 12558. 10.1038/ncomms12558. PMC4996975 27552116

[ece371154-bib-0075] Voigt, C. C. , M. Krofel , V. Menges , B. Wachter , and J. Melzheimer . 2018. “Sex‐Specific Dietary Specialization in a Terrestrial Apex Predator, the Leopard, Revealed by Stable Isotope Analysis.” Journal of Zoology 306: 1–7. 10.1111/jzo.12566.

[ece371154-bib-0076] West, J. B. , G. J. Bowen , T. E. Cerling , and J. R. Ehleringer . 2006. “Stable Isotopes as One of nature's Ecological Recorders.” Trends in Ecology & Evolution 21: 408–414. 10.1016/j.tree.2006.04.002. 16753238

[ece371154-bib-0077] Zheng, Z. , Y. Xu , J. Wang , et al. 2019. “Environmental Stress and Eutrophication in Freshwater Wetlands: Evidence From Carbon and Nitrogen Stable Isotopes in Cattail (*Typha domingensis* Pers.).” Ecological Processes 8: 1–8. 10.1186/S13717-019-0186-4/TABLES/4.

