# Supplementary material for: The Cat's Whiskers: Stable Isotopes Reveal Individual Specialisation of Adaptable Caracals (Caracal caracal) Foraging in an Urbanising Landscape
Source: Ecol Evol. 2025 Mar 23;15(3):e71154. doi: 10.1002/ece3.71154 (PMC11930547; doi:10.1002/ece3.71154)
Supplement: Supplementary file 1 — Appendix S1. Supporting Information. [file ECE3-15-e71154-s001.docx]

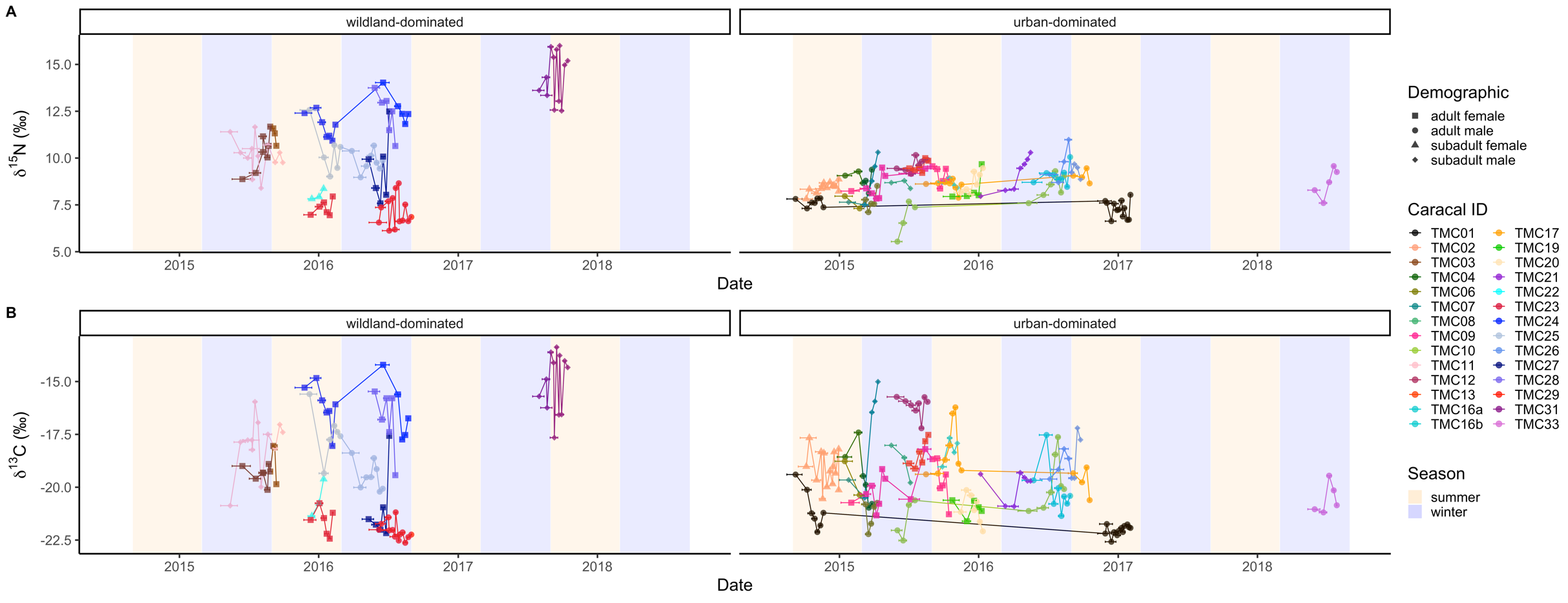
Supplementary material

**Fig. S1** Timeline plot of (A) δ^15^N and (B) δ^13^C values in caracal whiskers sampled from individuals with wildland- and urban-dominated home ranges on the Cape Peninsula, South Africa. Points are coloured by caracal individual and shaped by demographic group. Error bars represent upper and lower limits of date estimates based on growth rate estimates from Mutirwara et al (2018) [see *Methods*]. Shaded areas represent season (southern hemisphere winter vs summer).


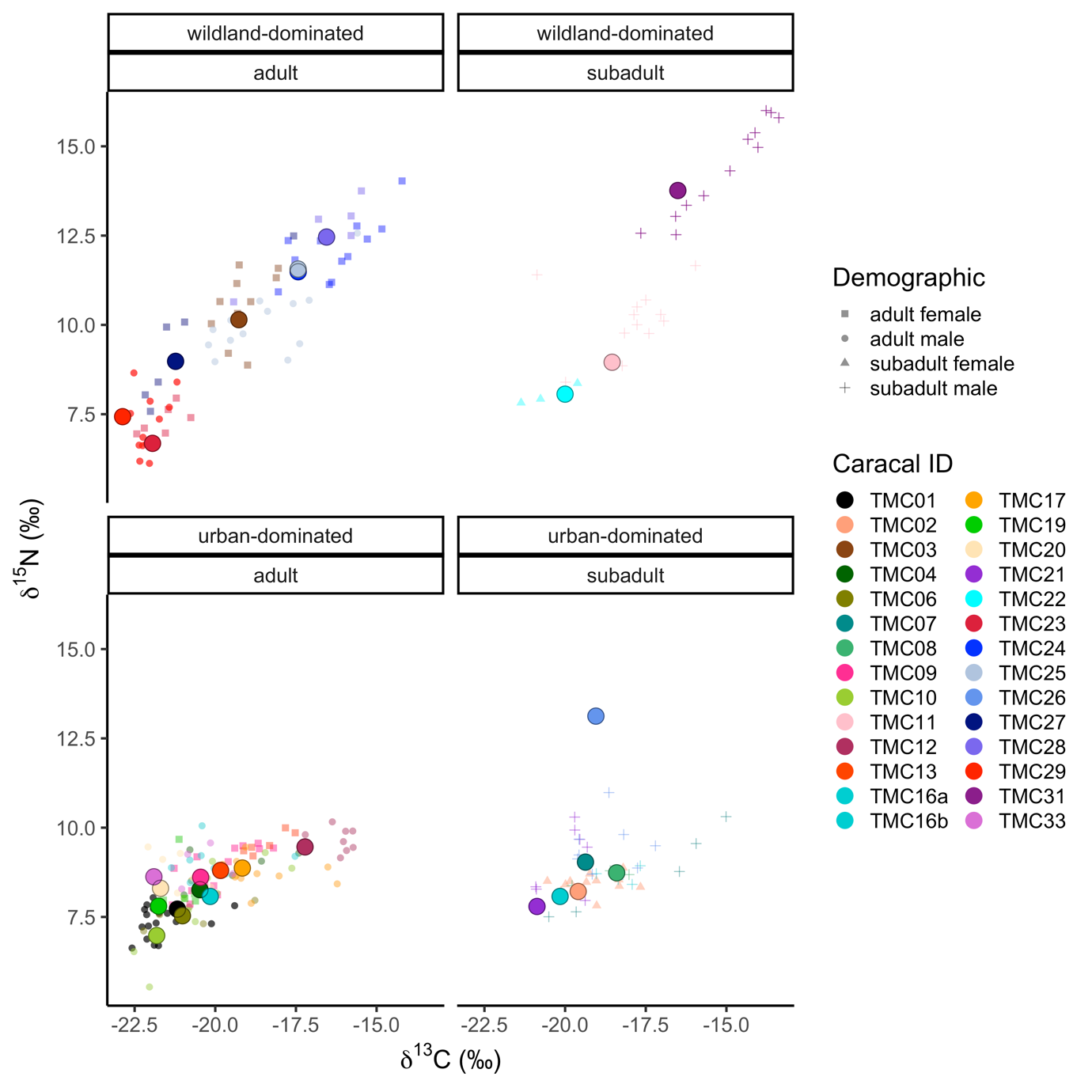


**Fig. S2** Isotope values in fur (large points) and whisker segments (small points) sampled from caracals with the wildland- and urban-dominated home ranges on the Cape Peninsula, South Africa. Fur points are coloured by caracal individual. Whisker points are coloured by caracal individual and shaped by demographic group. Fur and whisker isotope values are similar for all but one subadult male (TMC26), where the sampling period is different (fur from capture and whisker from necropsy).


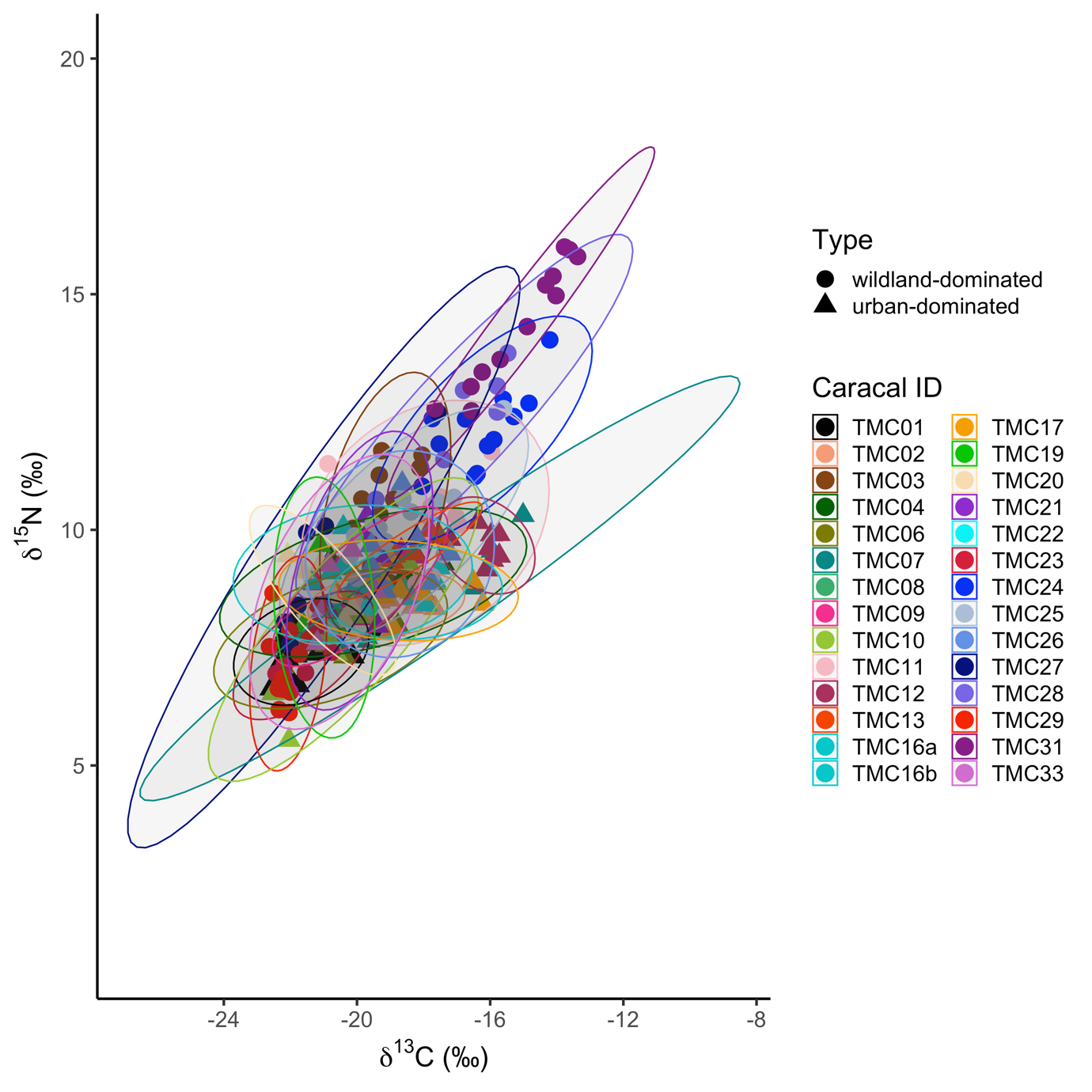


**Fig. S3** Bayesian ellipses of individual caracals (n = 28) sampled on the Cape Peninsula, South Africa

**Table S1** Full model structure for linear mixed models (LMMs) of δ^13^C and δ^15^N values investigating the potential influence of season and caracal groups (age, sex and urban- vs wildland-dominated home ranges), the effect of seasonal impacts on spatial variables, and the effect of seasonal impacts on dietary variables (six models in total)

| **Variable** | **δ^13^C / δ^15^N**  **season + caracal groups** | **δ^13^C / δ^15^N season + spatial** | **δ^13^C / δ^15^N season + dietary** |
| --- | --- | --- | --- |
| age (adult vs subadult) | X |  |  |
| sex (male vs female) | X |  |  |
| home range type (urban- vs wildland-dominated) | X |  |  |
| season (summer vs winter) | X |  |  |
| demographic (adult male, adult female, subadult male, subadult female) |  | X | X |
| season*proportion wetland area |  | X |  |
| season*proportion urban area |  | X |  |
| season*coastal distance |  | X |  |
| season*terrestrial biomass |  |  | X |
| season*arboreal biomass |  |  | X |
| season*marine biomass |  |  | X |
| season*wetland biomass |  |  | X |
| Random effect: caracal ID | X | X | X |
| Random effect: home range type |  | X | X |

**Table S2** Home range size for caracals (n = 28) on the Cape Peninsula, South Africa.

| **Sex** | **Age class** | **n** | **Mean (± SD) home range size (km^2^)** |
| --- | --- | --- | --- |
| female | adult | 9 | 16.83 ± 13.91 |
|  | subadult | 1 | 7.54 |
| male | adult | 11 | 64.54 ± 28.86 |
|  | subadult | 7 | 32.15 ± 36.62 |

**Table S3** Bayesian Layman metrics for all female and male caracals, adult and subadult caracals, and individuals with wildland-dominated and urban-dominated home ranges on the Cape Peninsula, South Africa.

| **Metric** | **Female** | **Male** | **Adult** | **Subadult** | **Wildland** | **Urban** |
| --- | --- | --- | --- | --- | --- | --- |
| _ncaracal_ | 10 | 18 | 19 | 9 | 10 | 18 |
| N_whisker segment_ | 83 | 161 | 176 | 68 | 94 | 150 |
| Range of δ^15^N | 5.06 | 7.23 | 5.24 | 6.35 | 7.23 | 2.26 |
| Range of δ^13^C | 5.36 | 7.00 | 5.93 | 5.51 | 7.00 | 5.54 |
| Total ellipse area | 8.54 | 15.31 | 12.80 | 8.68 | 9.76 | 4.78 |
| Centroid distance | 2.06 | 1.82 | 1.88 | 1.72 | 2.70 | 1.25 |
| Mean nearest neighbour distance | 0.87 | 0.75 | 0.56 | 1.27 | 0.95 | 0.46 |
| Standard deviation of nearest neighbour distance | 0.30 | 1.07 | 0.45 | 1.48 | 0.65 | 0.36 |
